# Metagenomic analysis of an ecological wastewater treatment plant’s microbial communities and their potential to metabolize pharmaceuticals

**DOI:** 10.12688/f1000research.9157.1

**Published:** 2016-07-28

**Authors:** Ian N. Balcom, Heather Driscoll, James Vincent, Meagan Leduc

**Affiliations:** 1 Department of Natural Sciences, Lyndon State College, Lyndonville, VT, USA; 2 Vermont Genetics Network, Department of Biology and Physical Education, Norwich University, Northfield, VT, USA; 3 Vermont Genetics Network, Department of Biology, University of Vermont, Burlington, VT, USA

**Keywords:** Wastewater treatment, microbial ecology, pharmaceuticals, micropollutants, metagenome, rhizoplane, eco-machine, biodegradation, biofilm

## Abstract

Pharmaceuticals and other micropollutants have been detected in drinking water, groundwater, surface water, and soil around the world. Even in locations where wastewater treatment is required, they can be found in drinking water wells, municipal water supplies, and agricultural soils. It is clear conventional wastewater treatment technologies are not meeting the challenge of the mounting pressures on global freshwater supplies. Cost-effective ecological wastewater treatment technologies have been developed in response. To determine whether the removal of micropollutants in ecological wastewater treatment plants (WWTPs) is promoted by the plant-microbe interactions, as has been reported for other recalcitrant xenobiotics, biofilm microbial communities growing on the surfaces of plant roots were profiled by whole metagenome sequencing and compared to the microbial communities residing in the wastewater. In this study, the concentrations of pharmaceuticals and personal care products (PPCPs) were quantified in each treatment tank of the ecological WWTP treating human wastewater at a highway rest stop and visitor center in Vermont. The concentrations of detected PPCPs were substantially greater than values reported for conventional WWTPs likely due to onsite recirculation of wastewater. The greatest reductions in PPCPs concentrations were observed in the anoxic treatment tank where *Bacilli* dominated the biofilm community. Benzoate degradation was the most abundant xenobiotic metabolic category identified throughout the system. Collectively, the microbial communities residing in the wastewater were taxonomically and metabolically more diverse than the immersed plant root biofilm. However, greater heterogeneity and higher relative abundances of xenobiotic metabolism genes was observed for the root biofilm.

## Introduction

The treatment of human wastewater by ecological systems predates the advent of engineered wastewater treatment plants (WWTPs). While exposure to human pathogens is greatly reduced in communities where modern wastewater treatment technologies have been implemented ^1^, widespread detection of micropollutants in the environment ^2^ raises serious concerns about the efficacy of modern WWTPs to treat this class of contaminants. Moreover, with about two-fifths of the world’s population experience health effects due to poor sanitary conditions ^3^. The emerging field of ecological engineering has provided a variety of viable, cost-effective wastewater treatment designs ^4^. The organizing principle of ecological wastewater treatment is the construction of “task oriented mesocosms” ^5, 6^ of eutrophic ecosystems that, like conventional systems, primarily rely on microbial metabolic processes to achieve water quality goals. Where ecological WWTPs and conventionally engineered WWTPs differ significantly is in their reliance on ecological processes to assimilate nitrogen, phosphorous, and carbon from the wastewater into biomass. Whereas, conventional WWTPs utilize mechanically assisted, microbial processes to evolve gaseous C, N, and frequently chemical precipitation of phosphorous. A number of ecological systems have been operating around the world for decades including constructed wetlands ^7, 8^, Eco-Machines ^TM^ ^4^, and biofilters ^9, 10^ for residential, industrial, and municipal wastewater. These systems perform reliably based on tertiary wastewater standards ^4^, while reducing operational costs ^11^ and environmental and human health impacts of wastewater ^12^. The unique potential provided by ecologically engineered waste management is direct conversion of a liability (i.e. wastewater) to an asset (sequestered carbon, biomass, products, biodiversity, etc.) ^13^.

At the core of wastewater treatment is the biodegradation, oxidation, and reduction of organic macromolecules and inorganic chemical species primarily by resident microbial communities. While the microbial communities of conventional WWTPs have been thoroughly studied ^14^, very little is known about microbial communities in existing ecological WWTPs despite the fact that they are central to the functions these systems provide ^6^. The introduction of activated sludge and environmental media from diverse sources is thought to provide essential microbial functional groups ^4, 15^. It is not known whether these “seeding” events provide microbial functional groups with the capacity for biodegradation of micropollutants.

The promotion of microbial biodegradation of recalcitrant xenobiotic pollutants by plant roots has been well documented ^16, 17^. The “rhizosphere effect” ^18– 20^, driven by the release of plant metabolites from plant roots, accelerates microbial biodegradation of recalcitrant pollutants in soil and water ^21, 22^. In some cases, microbial biodegradation is promoted by a nonspecific increase in microbial metabolic activity in the area surrounding roots ^23^, yet other studies have shown a relationship between specific plant metabolites and certain pollutant degrading organisms ^24^. The interaction has been described as co-metabolic induction, or “co-metabolism”, where metabolism for one compound is promoted in the presence of other compounds ^21, 22^. This phenomenon has been successfully employed to accelerate the removal of a variety of recalcitrant pollutants from the soil and water including polychlorinated biphenols (PCBs) ^19, 20, 24^, polycyclic aromatic hydrocarbons ^22^, and chlorinated solvents such as trichloroethylene ^25^. However, little research has been done on whether co-metabolism is occurring in ecological WWTPs as a result of plant-microbe feedback processes. Using whole metagenome sequencing (WMS) we have examined whether the microbial populations residing on the plant roots immersed in wastewater of an ecological WWTP showed evidence of the capacity for micropollutantant biodegradation. These populations were compared to microbial communities free-floating in the wastewater, enrichment cultures growing on individual pharmaceutical compound carbon sources, as well as PPCP concentrations throughout the treatment system to determine whether plant-microbe feedback processes are supporting PPCP biodegradation in ecological WWTPs.

## Materials and methods

### Materials

Carbamazepine (5 *H*-Dibenz[ *b*, *f*]azepine-5-carboxamide), sulfamethoxazole (4-Amino- *N*-(5-methyl-3-isoxazolyl)benzenesulfonamide), and trimethoprim (2,4-Diamino-5-(3,4,5-trimethoxybenzyl)pyrimidine) were purchased from Sigma Aldrich.

### EcoMachine ^TM^ sampling

**Figure 1.**
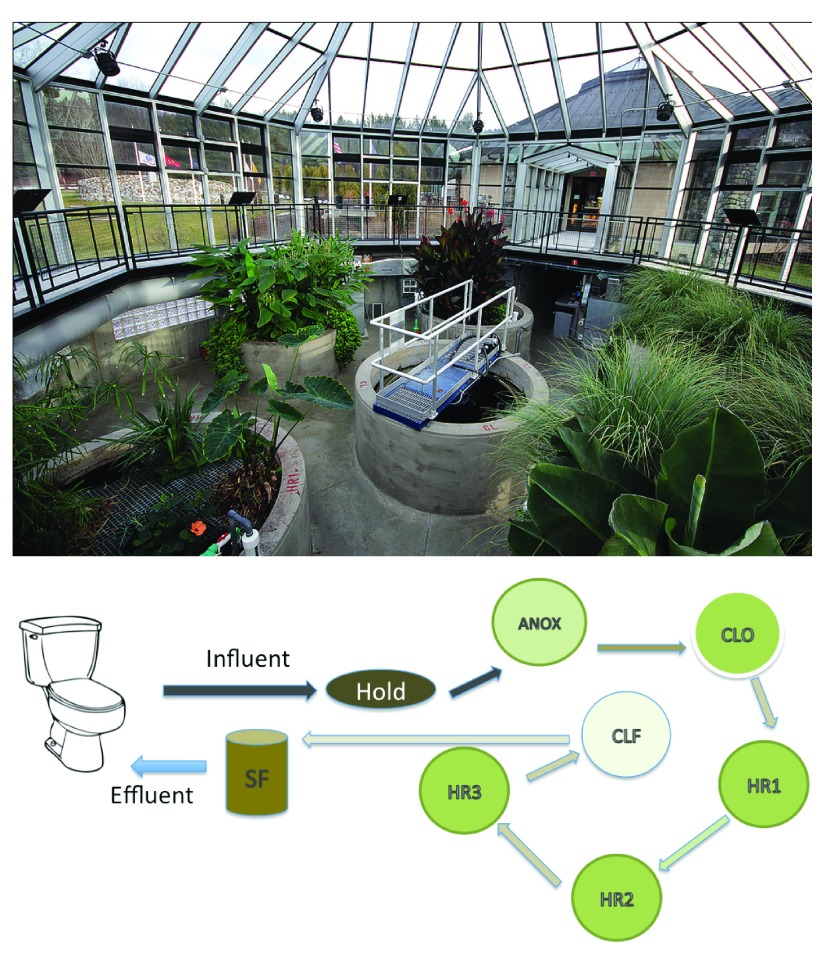
System layout of the Vermont, Vietnam Memorial and Visitor Center ecological WWTP. Abbreviations: Hold – holding tank, ANOX- anoxic tank, CLO- closed tank, HR1, HR2 and HR3- planted aerobic tanks, CLF- clarifier, SF sand filter.

An ecological WWTP (Eco-Machine ^TM^) in Sharon, Vermont at the Vietnam Memorial rest area on northbound Interstate 89 (43.727896, -72.425564) was sampled on June 30 and July 1, 2013. Wastewater from the toilets, urinals, and sinks, is collected in a holding tank and then treated in a series of tanks ( Figure 1). These consist of an anoxic tank (ANOX), a closed tank (CLO) and planted aerobic tanks (HR1, HR2 and HR3). These are followed by a clarifier and final treatment by a sand filter (SAND). The treated water (effluent, hereafter) is disinfected with the addition of sodium hypochlorite and dyed blue prior to returning to the toilets and urinals for reuse. To accommodate the approximately 48 hour residence time of the wastewater in the system [personal communication-Phil Gates, Simon Management Services], samples of aqueous phase and the immersed biofilm were collected from the first three tanks (ANOX, CLO, HR1) on June 30, 2013 and the latter three tanks (HR2, HR3, and SAND) on July 1, 2013. Plant root biofilm samples consisted of multiple roots from each individual tank composited into one sample. Influent wastewater samples (INF) were collected from the holding tank on June 30 ^th^.

### PPCP quantification

Duplicate 1 L aqueous phase samples were collected from each of the treatment tanks as well as the system INF and effluent (EF) for quantification of PPCPs by EPA method 1694 ^26, 27^ at a commercial analytical lab (TestAmerica, Sacramento, CA) using the Waters Acquity UPLC System and Waters Micromass Quattro Premier XE Mass Spectrometer.

### Enrichment cultures

Enrichment cultures with the pharmaceutical compounds carbamazepine, trimethoprim, and sulfamethoxazole (0.1M) serving as individual carbon sources were initiated using wastewater effluent inoculum in 100 mL carbon-free mineral salts medium (10 mM KH _2_PO _4_, 3 mM NaH _2_PO _4_, 1 mM MgSO _4_, 1mM NH _4_SO _4_ and trace minerals ^28^). Carbamazepine was delivered with minimal amounts of methanol added to the flask immediately after autoclaving and was allowed to evaporate leaving small suspended crystals as the sole carbon source. Starting with 1 mL of the WWTP sample, enrichment cultures were maintained at room temperature in a rotary shaker (100 rpm) for approximately 90 days. Five replicate cultures were initiated for each individual pharmaceutical carbon source.

At the third serial enrichment samples from the carbamazepine cultures (C3A, C3B, and C3D), trimethoprim cultures (T3B, T3C, and T3D) and sulfamethoxazole cultures (S3B, and S3D) were selected based on visual verification of microbial growth in the flasks. These eight samples were used for all further analyses.

### Genomic DNA extraction

Total genomic DNA was extracted and combined from duplicates for all samples using the following methods: water, biofilm, and enrichment culture samples were centrifuged at > 8,000 g for 1 min. Excess liquid was removed and pellets containing microbial samples were homogenized. Homogenization was performed using ~300 mg of a 50/50 mix of 1 mm and 100 μm AlO _3_ abrasive and 1 1/4 mm ceramic ball (Matrix F equivalent-MP Biomedical) and FastPrep-24 (MP Biomedicals, Santa Ana, CA) for 20 sec. at 6.5 R/S. 10 μL of 10 μg/μL lysozyme (Sigma), 4 μL of 400 U/μL Achromopeptidase (Sigma), 2 μL Mutanolysis (5U/μL) prepared in 10 mM TRIS buffer were added to each sample, which were briefly vortexed and incubated overnight at 37°C. The samples were then extracted using the standard method outlined by the E.Z.N.A. ^®^ Mollusc DNA isolation kit (Omega-Biotek, Inc, Norcross, GA), and the resulting DNA was quantified and its quality was assessed using the Nanodrop spectrophotometer (Thermo Scientific, Madison, WI), and Qubit Spectrofluorometer (Life Technologies, Carlsbad, CA) according to manufacturer’s instructions. After duplicate samples were combined, the resulting DNA concentrations were between 1.1 ng/μL for the wastewater samples obtained from tanks HR1 and HR2 and 17.9 ng/μL for the biofilm sample collected from the anoxic tank. Fragmentation of 10–100 ng of the resulting DNA was performed using a Covaris S2 AFA sonicator (Covaris Corp., Woburn, MA) equipped with MicroVails ( http://covarisinc.com/products/afa-tubes-and-vials/microtube-15/) to yield a size range of 200–500 bp as confirmed through a high sensitivity microfluidic DNA chip on the Bioanalyzer 2100 (Agilent Technologies, Santa Clara, CA) according to the manufacturer's instructions. The Agilent 2100 Bioanalyzer is an automated microfluidic-chip that is widely used to assess the DNA size fragment distribution and quantification in next-generation sequencing.

### Illumina ^® ^library preparation

Library preparation was performed using 45 ng of DNA (except samples HR1_W and HR2_W, which produced a total of 33 ng of DNA) in accordance with the Illumina ^®^ TruSeq DNA Sample Prep LT version 2 SOP (Part # 15026486 Rev. C, July, 2012) with the indicated reagents (DNA kit #FC-121-2001). According to manufacturer’s instructions, each sample was subjected to end repair, adenylation, and ligation of Illumina adaptors for indexing purposes. PCR amplification was performed using Illumina reagents (Part#15012995) followed by quantification using the Qubit spectrofluoromter and qPCR quantitation kit (KAPA Biosciences kit # 4824). Library quality and insert size distribution was assessed using the Agilent Bioanalyzer 2100.

### Massively parallel sequencing

Cluster generation and paired-end sequencing were performed at the Delaware Biotechnology Institute (DBI), University of Delaware, using an eight lane high-capacity v3 flowcell on the Illumina cBOT and HiSeq 2000 sequencer (Illumina, San Diego, CA), respectively. The WWTP samples (twelve) and the enrichment culture samples (eight) were multiplexed and run on two lanes. DBI delivered 20 FASTQ files with raw sequence data.

### Sequence processing

Raw sequences were checked for quality with FastQC v0.10.1 ( http://www.bioinformatics.babraham.ac.uk/projects/fastqc/). Trimmomatic v0.30 ^29^ was used to remove adapters and filter low-quality base calls/reads. Leading and trailing bases below quality 20 and reads less than 40 bases in length were removed. Additionally, reads were scanned using a 5-base wide sliding window and cut when the average quality per base dropped below 20. PhiX Control v3 from Illumina was used as a low-concentration spike-in during sequencing at DBI. Quality-trimmed FASTQ files were aligned to the PhiX genome (NCBI RefSeq NC_001422.1) using Bowtie2 2.2.3 ^30^ and all aligned reads were removed. Quality-trimmed and filtered reads were verified with FastQC prior to taxonomic and functional characterization.

### Nucleotide sequence accession numbers

All twenty FASTQ files and associated metadata are available through NCBI BioProject ID PRJNA286671 ( http://www.ncbi.nlm.nih.gov/bioproject/286671).

### Bioinformatic analysis

Translated trimmed reads served as input for a protein-level homology search against NCBI-NR, ( ftp://ftp.ncbi.nlm.nih.gov/blast/db/FASTA/nr.gz, downloaded May 26, 2015) a comprehensive non-redundant protein database, using the BLAST-like tools RAPSearch2 v2.16 ^31^ for WWTP samples and DIAMOND v.0.7.9 ^32^ for enrichment culture samples. DIAMOND was used instead of RAPSearch2 for analysis of enrichment culture samples because it was designed to easily integrate with MEtaGenome ANalyzer (MEGAN). It implements an algorithm that is similar to, but faster than, RAPSearch2, it was newly available when the enrichment cultures’ sequence data was ready for analysis, and control sample testing showed nearly identical taxonomic profiles from DIAMOND as those generated with RAPSearch2 searches.

The similarity search results for each sample set, which include all reads with alignments to the NR protein database and their GI accession numbers (maximum 25 alignments per read) were imported separately into MEGAN v5.7.10 ^33^ ( http://ab.inf.uni-tuebingen.de/software/megan5/). MEGAN parsed the RAPSearch2 (WWTP) and DIAMOND (enrichment culture) results using the lowest common ancestor (LCA) algorithm ^34^ and NCBI taxonomy ( ftp://ftp.ncbi.nlm.nih.gov/pub/taxonomy/taxdmp.zip downloaded March 26, 2015) (lowest common ancestor parameters: maxMatches=100 minScore=50.0 maxExpected=1.0 topPercent=10.0 minSupportPercent=1.0 minSupport=50 minComplexity=0.44). Reads that passed this filter and that were unambiguously assigned to a NCBI taxon by LCA were retained in each sample’s MEGAN results file. A combined MEGAN file was generated for WWTP samples, as well as for enrichment culture samples, with read counts normalized to the sample with the fewest input reads in each set.

Two positive controls were used to validate our bioinformatics pipeline and to establish a minimum support threshold (or false-positive cut-off) for taxonomic profiling. One control dataset is comprised of single-end Illumina reads from a synthetic microbial sample prepared by CosmosID. This constructed freshwater sample simulates organisms found in the Delaware River and is described here: http://www.cosmosid.net/constructed-freshwater. The second control dataset is single-end Illumina reads from the Human Microbiome Project (HMP) mock community even sample ( http://www.ncbi.nlm.nih.gov/sra/SRX055380). Reads from both positive control samples can be downloaded from BaseSpace: https://basespace.illumina.com/projects/20039022/samples. 

Using MEGAN, reads were annotated based on the KEGG (Kyoto Encyclopedia of Genes and Genomes) functional classification of enzymes and pathways ^36^. Using auxiliary index files obtained from the MEGAN website (gi2kegg.map.gz, built Dec 1, 2010), GI accession numbers were mapped to KEGG functional groups based on the highest MinScore match (minimum MinScore = 50). Reads may be assigned to more than one functional group per classification system, as each KEGG group may appear in several functional categories.

The relatedness of the microbial communities located in the different tanks and phases of the WWTP was assessed through pairwise similarity scores computed in MEGAN using a normalized Goodall’s probabilistic similarity index ^38^ for both phylogenetic and metabolic profiles for each sample. Graphical representations of the distance matrices were generated in MEGAN as un-rooted phylogenetic neighbor networks ^39^. A Venn diagram was produced (Partek ^®^ Genomics Suite ^®^ software, version 6.6 build 6.15.1016 Copyright; 2014, Partek Inc., St. Louis, MO, USA) to illustrate taxa common to the different sample datasets.

Statistical Analysis of Metagenomic Profiles (STAMP) software v2.1.3 ^40^ was used to test statistical significance of differentially abundant taxonomic groups and functional categories for 1) WWTP sample groups (aqueous and biofilm phases) and 2) enrichment culture sample groups (carbamazepine (C), sulfamethoxazole (S), and trimethoprim (T)). LCA taxonomic profiles and KEGG including abundances, were imported to STAMP for each sample set. Two-sided Welch's t-test was used to compare aqueous and biofilm phases with a confidence interval of the effect size and multiple test correction using the Benjamini-Hochberg FDR method. One-way ANOVA was used to compare enrichment culture groups with an effect size (Eta-squared) and multiple test correction using the Benjamini-Hochberg FDR method. Tukey-Kramer post-hoc test (0.95) was used to determine which means were significantly different when an ANOVA produced a significant p-value.

To visualize the distribution of microbial taxa in WWTP samples the Circos software package v0.69 ^41^ was used to depict the location and relative abundances of microbial taxa at the class rank identified in MEGAN using the LCA algorithm.

## Results

### PPCPs concentrations

There were 11,568 visitors during the week in which sampling was conducted (June 25–July 1, 2013). Each visitor used an average of 2.27 liters of water contributing 26,452 liters of water to the wastewater treatment system ^42^. The wastewater used to isolate microbial DNA samples contained detectable concentrations of caffeine, carbamazepine, DEET, gemfibrozil, ibuprofen, naproxen, sulfamethoxazole, thiabendazole, and trimethoprim. Of the compounds detected in influent water (from facility toilets, urinals, and sink drains), the cholesterol medication gemfibrozil was detected at the highest concentration (1.5 × 10 ^5^ ng L ^-1^), followed by caffeine, ibuprofen, and naproxen (9.5, 6.6, and 5.5 × 10 ^4^ ng L ^-1^, respectively) ( Table 1). The concentrations of PPCPs in the wastewater samples generally decreased the further through the treatment process ( Figure 1) the sample was obtained. However, gemfibrozil, caffeine, and ibuprofen were detected at higher concentrations in the sand filter or effluent water samples than the preceding tank. The concentrations of carbamazepine, DEET, and trimethoprim did not change substantially over the entire treatment process.

**Table 1.** Concentrations of detected pharmaceutical compounds in the ecological wastewater treatment plant. Concentrations (ng L ^-1^) of detected pharmaceuticals and personal care products in the wastewater sampled from each major treatment tank of the WWTP. Abbreviations: INF- influent, ANOX- anoxic closed tank, CLO-closed aerobic tank, HR1, HR2 & HR3- planted aerobic tanks, SF- sand filter, EF- fffluent, Caff- caffeine, Carb- carbamazepine, DEET - *N,N*-Diethyl-3-methylbenzamide, Gemf- gemfibrozil, Ibup- ibuprofen, Napr- naproxen, Sulf- sulfamethoxazole, Thia- thiabendazole, Trim- trimethoprim, ND-not detected above method reporting limit.

	Caff	Carb	DEET	Gemf	Ibup	Napr	Sulf	Thia	Trim
INF	95000	ND	ND	150000	66000	55000	7700	12000	ND
ANOX	19000	770	640	3500	11000	6500	2900	ND	550
CLO	4800	730	620	3600	2100	3000	2000	ND	290
HR1	1300	590	540	1100	560	1600	1100	ND	450
HR2	602	540	520	890	300	540	980	ND	420
HR3	510	530	550	770	ND	ND	930	ND	480
SF	1000	590	540	ND	ND	ND	960	ND	490
EF	570	550	560	2400	340	ND	860	ND	460

### Metagenome sequencing and sequence processing

 Whole metagenome shotgun sequencing of 12 WWTP samples generated more than 388 million paired-end reads, 101 bp in length, with an average depth of 32.4 million reads per sample (range: 2.35–53.3 million) ( Supplementary Material ST13). Eighty-eight percent of raw reads (343,355,560) were retained after quality-trimming and were aligned to the NCBI-NR protein database. Of the 175,238,945 reads with at least one hit to NR proteins, approximately 84% were assigned taxonomy by the lowest common ancestor (LCA) algorithm in MEGAN. Over half of quality-trimmed reads in our samples (51%) had no protein hits in NCBI-NR and 19.3% of reads with protein hits could not be classified by the LCA algorithm. As a result, the latter were designated “Not Assigned” reads in MEGAN.

Whole metagenome shotgun sequencing of the 8 enrichment culture samples generated more than 177 million paired-end reads, 101 bp in length, with an average depth of 22 million reads per sample (range: 17–29 million) ( Supplementary Material ST14). Eighty-nine percent of raw reads (157,598,266) were retained after quality-trimming and were aligned to the NCBI-NR protein database. Of the 96,824,600 reads with at least one hit to NR proteins, over 99% were assigned taxonomy by the LCA algorithm in MEGAN. Nearly 39% of quality-trimmed reads in our samples (60,773,666) had no protein hits in NCBI-NR and 0.4% of reads with protein hits could not be classified by the LCA algorithm and were designated “Not Assigned“ reads in MEGAN. The minimum-support percent threshold in MEGAN for both WWTP and enrichment culture analyses was set to 1.0% based on our bioinformatics workflow results from the HMP and Delaware River control samples.

### Taxonomic classification of sequences

**Figure 2.**
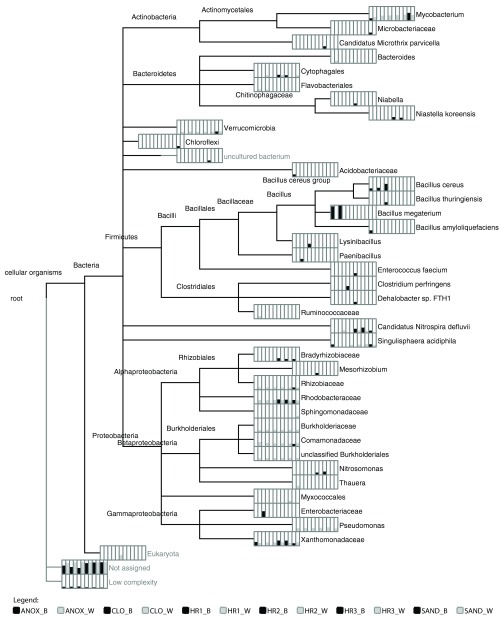
Taxonomic composition and relative abundance of the ecological wastewater treatment plant’s microbial communities. Phylogram depicting the lowest common ancestor taxonomic composition of the ecological wastewater treatment plant. Bar chart for each taxon (depicted in the order shown in the legend) indicate the number of reads (normalized) associated with each taxonomic classification, shown here in square-root scale to highlight differences. Wastewater treatment plant sample locations with _W and _B indicate aqueous and immersed biofilm samples, respectively. Abbreviations: ANOX- anoxic tank, CLO- closed aerobic tank, HR1, HR2 and HR3- planted aerobic tanks, and SAND - sand filter.

**Figure 3.**
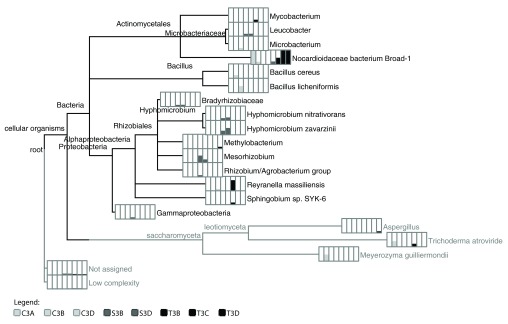
Taxonomic composition and relative abundance of the pharmaceutical-degrading liquid enrichment cultures. Phylogram depicting the lowest common ancestor taxonomic composition of the sole pharmaceutical compound carbon source enrichment cultures. Bar charts for each taxon (depicted in the order shown in the legend) and indicate the number of reads (normalized) associated with each taxonomic classification, shown here in square-root scale to highlight differences. Samples starting with C, T, and S indicate sequences obtained from cultures grown on carbamazepine, trimethoprim, and sulfamethoxazole carbon sources, respectively.

The LCA algorithm provided a microbial taxonomic profile of the 12 WWTP samples and the 8 enrichment culture samples ( Figure 2 and Figure 3, respectively). The read counts were normalized to the sample with the least number of total reads to allow relative abundance to be depicted and shown as bar-charts at the leaves of each phylogram. The terminal taxa from various ranks identified by the LCA algorithm in the 12 WWTP samples ranged from a low of 8 taxa (HR1_B sample) to 17 taxa (ANOX_W sample) ( Figure 2; Supplementary Material ST2). Members of *Mycobacterium, Pseudomonas,* and Verrucomicrobia were identified in all of the aqueous phase (_W) samples as well as the biofilm in the anoxic tank (ANOX_B) and the sand filter (SAND_B). Additionally, the families Rhodobacteraceae, Burkholderiaceae, Comamonadaceae and Xanthomonadaceae were identified in all of the aqueous phase samples as well as in at least one, but not all immersed biofilm samples. The family Xanthomonadaceae was identified in 10 of the 12 WWTP samples, followed by Rhodobacteraceae (9), Comamonadaceae and Rhizobiaceae (7), and Burkholderiaceae (6).

According to the LCA algorithm taxonomic assignments by MEGAN, the enrichment cultures originating from the wastewater effluent inoculant produced mixed cultures ranging from 2 (T3B and T3C) to 8 (S3D) taxa identified in each culture ( Figure 3; Supplementary Material ST4). Bacteria were identified in all enrichment cultures and ascomycete fungi were in all carbamazepine cultures and one trimethoprim culture (T3D). The family Nocardioidaceae in the Propionibacterineae was identified in all but two of the cultures (C3D and S3B). Proteobacteria were identified in all but one of the carbamazepine cultures (C3B). Alphaproteobacteria was identified in two carbamazepine cultures (C3A and C3D), all sulfamethoxazole cultures as well as one of the trimethoprim cultures (T3D). Gammaproteobacteria was identified in one sulfamethoxazole culture (S3B). Numerous taxa were identified at the species level by LCA in MEGAN, including *Bacillus cereus, B. lichenfomis, B. subtilis, Clostridium perfringens, Hyphomicrobium denitrificans, H. nitrativorans, H. zavarzinii, Sphingobium* sp. SYK-6, *Trichoderma atroviride, T. virens,* and *Meyerozyma guilliermondii*.

The carbamazepine cultures (C3A, C3B, and C3D) all contained members of Actinomycetales, Ascomycete fungi, while Firmicutes and Alphaproteobacteria are represented in two of three replicates (C3B, C3D and C3A, C3D, respectively). Similarly, the trimethoprim enrichment cultures (T3B, T3C and T3D) contained Actinomycetes and Proteobacteria with Alphaproteobacteria and ascomycete budding yeast fungus *Aspergillus* in culture T3D only. The sulfamethoxazole enrichment cultures contained highest taxonomic richness with eight taxa identified by LCA in MEGAN in one of the replicates (S3D) and seven taxa in the other (S3B). These taxa collectively included members of the Actinomycetales ( *Leucobacter*), Rhizobiales (Bradyrhizobiaceae, *Hyphomicrobium, Mesorhizobium, Rhizobium/Agrobacterium* group), and Gammaproteobacteria.

**Figure 4.**
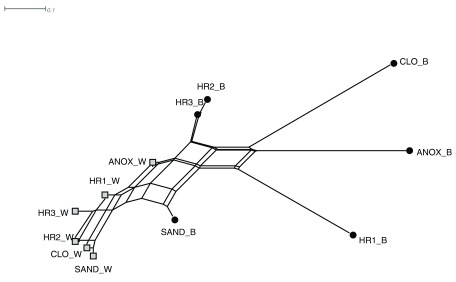
Taxonomic structure of the ecological wastewater treatment plant’s microbial communities. Neighbor-net depicting the taxonomic pairwise similarity (Normalized Goodall) of the lowest common ancestor of translated sequences obtained from the six major tanks of the WWTP and the two major phases (aqueous _W and immersed biofilm _B) of the ecological wastewater treatment plant. Abbreviations: ANOX - anoxic tank, CLO - closed aerobic tank, HR1, HR2 and HR3 - planted aerobic tanks, and SAND - sand filter.

A graphical representation of the pairwise distance matrix ^39^ generated using normalized Goodall’s similarity index ^38^ of the 12 WWTP samples is shown as an unrooted phylogenetic neighbor network in Figure 4. The LCA taxonomic assignments of the microbial populations residing in the aqueous phase samples cluster near one another in the neighbor network, while the immersed biofilm samples showed greater dissimilarity.

**Figure 5.**
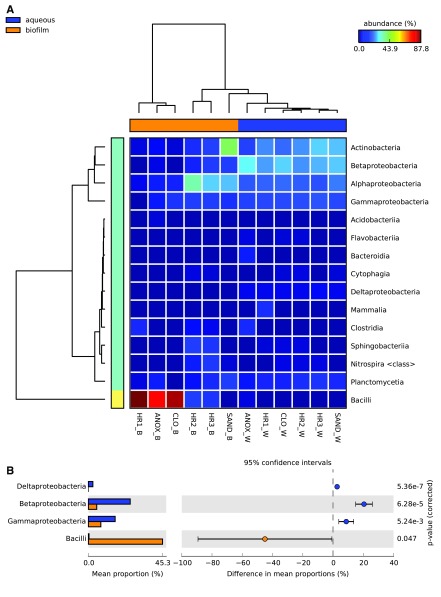
Relative abundance of microbial classes in the ecological wastewater treatment plant. **A**. Heat map depicting the relative abundance of microbial taxonomic classes identified in the different aqueous and biofilm phases in each treatment tank of the ecological wastewater treatment plant. **B**. Differences in mean proportions of major microbial taxonomic classes identified collectively in the aqueous and biofilm phases of the ecological wastewater treatment plant. Statistical Analysis of Metagenomic Profiles (STAMP) software v2.1.3. Abbreviations: W – aqueous, B – immersed biofilm, ANOX – anoxic tank, CLO – closed aerobic tank, HR1, HR2 and HR3 – planted aerobic tanks, and SAND – sand filter.

In STAMP, the aqueous and biofilm samples were compared at different taxonomic ranks to highlight differences between the WWTP sample sets. Figure 5 depicts the percent relative abundances of microbial classes identified in the 12 WWTP samples. In general, Actinobacteria, Alphaproteobacteria, Betaproteobacteria, and Gammaproteobacteria had higher relative abundances in the aqueous samples, whereas Bacilli were more abundant in the biofilm samples. The relative abundances of Bacilli in the biofilm samples were highest in the first three tanks (ANOX, CLO, HR1) and sharply reduced thereafter ( Figure 5a, Figure 9). The relative abundances of Deltaproteobacteria, Betaproteobacteria, Gammaproteobacteria, and Bacilli were significantly different between the two physical phases (aqueous and biofilm) sample sets ( Figure 5b).

**Figure 6.**
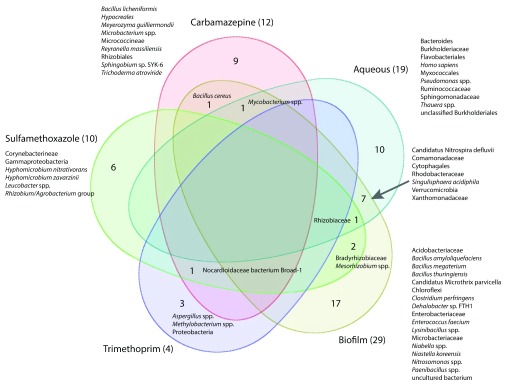
Co-occurrence of microbial taxa in the aqueous, biofilm, and pharmaceutical enrichment cultures. Venn diagram illustrating sequences with taxonomic identities retrieved from the aqueous and biofilm samples obtained from the ecological wastewater treatment plant and the carbamazepine, sulfamethoxazole and trimethoprim aqueous enrichment cultures. Numbers in parentheses after sample set names indicate taxa identified to species level in each metagenome sequence dataset. Numbers within the diagram indicate taxa common to the individual and overlapping datasets. (Partek ^®^ Genomics Suite ^®^ software, version 6.6 build 6.15.1016 Copyright 2014; Partek Inc., St. Louis, MO, USA)

To assess similarities in taxonomic composition between the pharmaceutical compound enrichment cultures and the WWTP samples, we examined the co-occurrence of LCA taxa among datasets ( Figure 6). The taxonomic similarities of the five major sample types were examined by combining all aqueous samples, all immersed biofilm samples, and the replicates of each pharmaceutical carbon source enrichment cultures into five datasets. As is typical of enrichment cultures originating from complex environmental samples, taxonomic similarities were limited to a few shared taxa. When combined, twelve microbial taxa were identified in the carbamazepine enrichment cultures. However, only three taxa were identified in at least two out of three replicate carbamazepine cultures. Of the twelve taxa identified in the combined carbamazepine cultures, only one each was identified in the biofilm and aqueous samples ( *Bacillus cereus*, *Mycobacterium* spp., respectively) obtained from the wastewater treatment plant. *Bacillus cereus* was identified in the biofilm samples obtained from the first three tanks of the wastewater treatment system (ANOX_B, CLO_B, and HR1_B). It has been associated with human feces ^43^. One taxon (Nocardioidaceae bacterium Broad-1) was found in six out of eight total enrichment culture samples, including all three trimethoprim samples. Additionally, Proteobacteria, *Aspergillus* spp. and *Methylobacterium* spp. were unique to trimethoprim enrichment cultures. Nocardioidaceae bacterium Broad-1, found as a byproduct during the genome assembly of the fungus *Coccidioides* (NCBI BioProject accession number PRJNA48513), is of unknown origin. Of the ten taxa identified in the combined sulfamethoxazole cultures, 50% were in both sulfamethoxazole samples, three were identified in the biofilm samples (Rhizobiaceae (HR2_B, HR3_B and Sand_B) *,* Bradyrhizobiaceae (Sand_B), and *Mesorhizobium* spp. (HR2_B)), and one was found in the all of the aqueous samples (Rhizobiaceae) obtained from the wastewater treatment plant. Seven out of 40 WWTP taxa were shared among the aqueous and biofilm samples.

To assess the influence of the carbon sources on enrichment culture taxonomic composition, LCA-based taxonomic profiles of the enrichment culture replicates were compared in STAMP. The most numerous significant differences with strong effect sizes were found at the family level (see supplementary material ST35). The relative abundance of reads from Bradyrhizobiaceae, Hyphomicrobiaceae, Microbacteriaceae, and Rhizobiaceae were significantly greater in sulfamethoxazole cultures than in trimethoprim or carbamazepine cultures (with FDR corrected p-values of 2.98 × 10 ^-8^, 0.0274, 0.0199, 0.381, respectively).

### Microbial metabolism of PPCPs

**Figure 7.**
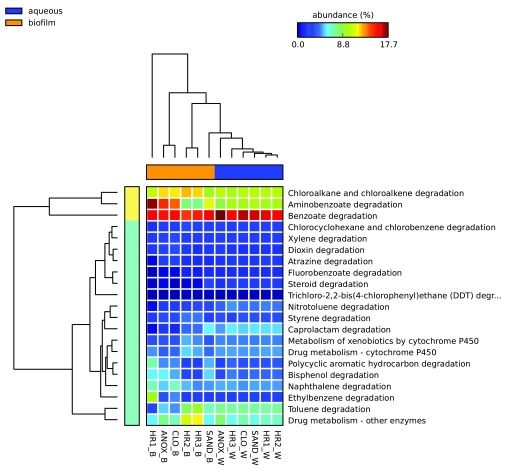
Relative abundance of xenobiotic metabolism genes identified in the microbial communities of the ecological wastewater treatment plant. Heat map indicating the relative number of sequence reads associated with the KEGG xenobiotic metabolism categories (on right) identified in each sample location and phase (on bottom). Statistical Analysis of Metagenomic Profiles (STAMP) software v2.1.3. Abbreviations: W – aqueous, B – immersed biofilm, ANOX – anoxic tank, CLO – closed aerobic tank, HR1, HR2 and HR3 – planted aerobic tanks, and SAND – sand filter.

To investigate the role of the WWTP’s microbial communities in the metabolism of PPCPs, KEGG was queried using MEGAN for sequences identified as involved in xenobiotic metabolism. Figure 7 depicts the percent relative abundances of sequences identified in the metagenomes obtained from each sample as involved in xenobiotic biodegradation and metabolism KEGG pathways for WWTP samples. The most abundant xenobiotic biodegradation and metabolism subcategory in all cultures and WWTP samples, with a few exceptions (HR1_B, C3B and C3D), was benzoate degradation comprising 14.5%–17.7% of reads in this subcategory for WWTP samples and 11.1%–23.6% in the enrichment culture samples. The biofilm sample obtained from the sand filter (Sand_B) at the end of the WWTP treatment train produced the greatest number of sequences (18,766) aligning to the “xenobiotic biodegradation and metabolism” category in the KEGG database followed by the aqueous samples of the sand filter (SAND_W) (18,318), and the aqueous phase of the third planted aerobic tank (HR3_W) (18,002). The sample with the greatest number of reads associated with KEGG pathway “drug metabolism - cytochrome P450” was the aqueous phase of the third planted aerobic tank (HR3_W) (1,384) followed by aqueous samples of the sand filter (SAND_W) (1,372) and the immersed biofilm sample obtained from the first planted aerobic tank (HR1_W) (1,330). The sample with the greatest number of reads associated with the KEGG category “drug metabolism – other enzymes” was the biofilm in the second planted aerobic tank (HR2_B) (2,181) followed by aqueous phase of the anoxic tank (ANOX_W) (1,904), and the biofilm sampled from the sand filter (SAND_B) (1,827).

**Figure 8.**
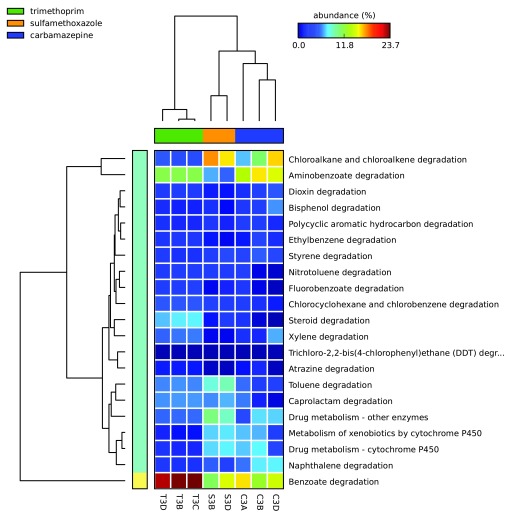
Relative abundance of xenobiotic metabolism genes identified in the pharmaceutical enrichment cultures. Heat map indicating the relative number of sequence reads associated with the KEGG xenobiotic metabolism categories (on right) identified in each pharmaceutical compound carbon source enrichment culture sample (on bottom). Samples starting with C, T, and S indicate metagenomes isolated from cultures grown on carbamazepine, trimethoprim, and sulfamethoxazole carbon sources, respectively. Statistical Analysis of Metagenomic Profiles (STAMP) software v2.1.3.

The percent relative abundances of xenobiotic metabolism-associated sequences identified in enrichment cultures’ metagenomes are illustrated as a heat map in Figure 8 (see Supplementary materials ST6). Reads associated with benzoate degradation were most abundant for trimethoprim cultures, whereas chloroalkane and chloroalkene degradation reads were most abundant for sulfamethoxazole cultures. The most abundant xenobiotic metabolism category for the carbamazepine cultures varied from culture to culture with aminobenzoate degradation, benzoate degradation, and chloroalkane/chloroalkene degradation categories for each of the three replicates. The samples C3A and T3D produced the greatest number of reads associated collectively with xenobiotic metabolism genes at 232, 201 and 218, 591, respectively. Of these, C3A and T3D metagenomes contained 10,151 and 11,889 reads associated with “drug metabolism – other enzymes” in KEGG, respectively. In this category, the sulfamethoxazole culture (S3B and S3D) had the most assigned reads at 16,102 and 13,730, respectively. For the category “Drug metabolism – cytochrome P450” the carbamazepine culture sample C3A contained the greatest relative number of sequences (16,199) followed by the sulfamethoxazole culture S3D (11,847).

## Discussion

To our knowledge, this is the first study to use WMS to characterize the microbial communities of an ecological WWTP. While a taxonomic description of the microbial communities is provided, here we have focused on microbial metabolism of PPCPs in the wastewater. Using WMS we have identified representation and relative abundance of microorganisms in the six major treatment tanks. To examine the role plants have on the structure of the microbial communities, we compared the communities found in the wastewater and those attached to plant roots immersed in the wastewater. Microbial metabolic pathways for most emerging pollutants, including micropollutants such as PPCPs, have not been characterized. Therefore, we have focused on the xenobiotic metabolic capacity as represented by the location and abundance of known genes represented in the KEGG database.

### PPCPs in the wastewater

The concentrations of PPCPs in the samples obtained from the Sharon, VT WWTP indicate that some of the PPCPs are being effectively removed by the system (naproxen and thiabendazole), while others are accumulating in the recirculating system (caffeine, carbamazepine, DEET, trimethoprim, and sulfamethoxazole) ( Table 1). The increasing concentrations of caffeine, gemfribozil and ibuprofen in or after the sand filter suggest partitioning to the aqueous phase from attenuated organic matter may be occurring in the sand filter. However, based on these data, biotic (biodegradation) and abiotic (partitioning to the primary sewage sludge) processes occurring in the influent holding tank and first treatment tank (ANOX) account for significant reductions in concentrations for the PPCPs gemfibrozil, naproxen, thiabendazole, and to a lesser degree caffeine, ibuprofen, sulfamethoxazole, whereas carbamazepine, DEET, and trimethoprim were not removed.

Partitioning to the solid phase (sewage sludge) is an important aqueous phase removal processes that is driven by a compound’s hydrophobicity ^44, 45^. For certain chlorinated organic compounds, the octanol-water partitioning coefficient (K _OW_) correlates positively with sorption to biosolids when log K _OW_ values range from 1.26 to 5.48 ^46^. Carbamazepine (log K _OW_ 2.3–2.5), DEET (log K _OW_ 2.18–2.44), and trimethoprim (log K _OW_ 0.9–1.4) show limited removal in the Sharon, VT WWTP ( Table 1). These findings are consistent with similar studies on partitioning of PPCP in conventional WWTP ^47– 49^. Partitioning rates from aqueous influent to biosolids (sewage sludge) is variable with log K _d_ (solid-water distribution) values ranging from <0.7 to 4.2 ^50^ depending on the compound. The removal of the parasiticide, fungicide thiabendazole (log K _OW_ 2.47) from the wastewater between the influent sample location and the anoxic treatment tank was likely due to strong partitioning to the primary sludge in the holding tank. With this exception, all other compounds were detected in more than one tank of the system. The aqueous concentrations of the cholesterol-lowering drug gemfribozil (log K _OW _4.77) also indicated strong partitioning to the primary sludge showing reduction in concentration by three orders of magnitude between the influent and the anoxic treatment tank.

Pharmaceuticals and PPCPs in wastewater can undergo a number of processes that contribute to their complete or partial aqueous phase removal in wastewater treatment systems ^45^. These include chemical and or physical processes such as sorption to organic matter ^46, 48, 51^, photolysis ^52, 53^, volatilization ^50^, and biological transformation ^54^. Biological transformation is unique among these as it provides WWTP operators the potential to increase the removal of PPCPs from wastewater while partially or completely mineralizing the compounds thereby eliminating any risks associated with their release to the environment. In contrast, sorption to biomass (primarily sewage sludge) results in decreased mineralization ^55^, which when applied to land (dominant disposal method of the processed sewage sludge) is likely a significant source of PPCPs in the environment ^56^. It is unclear whether thermal treatment and dewatering, as is commonly done to biosolids prior to land application, alters the mass of PPCPs in this media.

The influent concentrations of the PPCPs were in many cases an order of magnitude or greater than the concentrations reported in conventional WWTP ^49, 57^. The Sharon, VT ecological WWTP recirculates the effluent onsite as flush water (sterilized and dyed blue prior to being used as toilet and urinal flush-water). The recirculation and reuse of the effluent is likely to result in an additive or concentrating effect for compounds with low removal and/or partitioning rates. Additionally, the concentration of PPCPs in municipal wastewater is likely diluted by the mixing of non-human wastewater such as wash water, storm water (in combined sewer systems), industrial process water and a variety of other sources. While the higher concentration of PPCPs in this recirculating ecological WWTP may present elevated exposure risks to operators and the environment if materials are discharged, conventional WWTP, which do not recirculate wastewater, are likely to discharge greater mass of PPCPs per liter wastewater treated. Additionally, retaining the PPCPs in the WWTP through recirculation is preferential to releasing them into receiving water bodies.

The results reported here are initial findings on the removal of PPCPs from the wastewater processed by this system as the species and concentrations of detected PPCPs are likely to change with time, fluctuating with the changing population of visitors. Significant variability in PPCPs species and mass loading into the system is likely responsible for the non-detects ( Table 1 ND’s) of individual compounds detected later in the treatment train. As we staggered our sampling of the initial three and latter three treatment tanks by 48 hours to accommodate the residence time of the wastewater in the treatment system, it is reasonable to assume that the concentrations quantified here represent the flux of PPCPs through the WWTP. Therefore, changes in the concentration of individual PPCPs throughout the system are the result of biotic and abiotic aqueous phase removal processes. The trends observed here could be influenced by fluctuations in PPCPs inputs. These fluctuations are likely responsible for the non-detects observed in the holding tank (INF) for carbamazepine, DEET and trimethoprim, while these compounds were detected in the next treatment tank (ANOX).

### Microbial communities of the ecological WWTP

**Figure 9.**
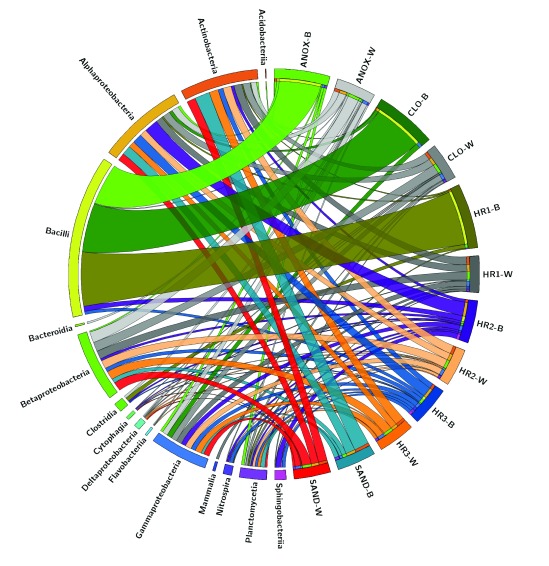
Location and relative abundance of microbial taxonomic classes identified in the two different phases of the ecological wastewater treatment plant. Relative abundances of microbial class-level taxa identified in the metagenomes isolated from the biofilm and wastewater phases of each treatment tank of the ecological wastewater treatment system. The relative abundance of each class in each sample is represented in the width of each ribbon. The clockwise order of the samples is represents the order of the wastewater treatment process. Circos software v0.69. Abbreviations: ANOX- anoxic tank, CLO- closed aerobic tank, HR1, HR2 and HR3- planted aerobic tanks, and SAND - Sand Filter.

The immersed biofilm and aqueous phase microbial communities exhibited two distinct taxonomic structures. According to the LCA algorithm, this difference was most evident at the class level ( Figure 5a). The relative abundances of Deltaproteobacteria, Betaproteobacteria, and Gammaproteobacteria were significantly higher in the microbial metagenomes of the aqueous phase, while Bacilli were observed in greater abundance in the immersed biofilm microbial communities ( Figure 5b). The relative abundance of Bacilli in the immersed biofilm communities was highly variable, with their dominance diminishing significantly after the first three tanks. Figure 9 illustrates the microbial taxonomic spatial variability of the ecological WWTP. The sample order in the diagram reflects wastewater movement through the treatment system starting with the anoxic tank (ANOX) moving clockwise to the sand filter (SAND). The width of each ribbon extending from the taxon to the sample represents relative abundance based on sequence counts. Bacilli dominated the immersed biofilm in the first three tanks (ANOX, CLO, and HR1), with representatives of other taxa present to a much lesser degree. The immersed biofilm samples also show greater microbial taxonomic richness in the latter phases of the treatment system (HR2, HR3, and SAND). This pattern is likely due to retention of fecal taxa by the earlier tanks as Bacilli are known to be abundant in human feces ^43^. Bacteroidia, which is also abundant in human feces ^58^, was not detected colonizing the immersed plant root surfaces. Only a small population was identified in the wastewater obtained from the anoxic tank.

Members of the Firmicutes (Bacilli) and Bacterioidia, which are common in human feces ^58^, were identified in the ecological WWTP microbial communities. However, these were the only organisms identified in the samples that are associated with human feces. Of the organisms used in water quality criteria to indicate contamination by feces (others include *Clostridium perfringens,* enterococci, *Escherichia coli*, and fecal coliforms), *Clostridium perfringens* was detected in only one sample, HR1_B ( Figure 2). This would indicate that the ecological WWTP is performing well with regard to attenuating fecal coliforms. Bacilli were dominant in the samples of immersed biofilm collected from the first three tanks in contrast to the taxonomic composition of the latter three treatment tanks ( Figure 2, Figure 9). This could indicate colonization of the surfaces in these initial treatment tanks by organisms of fecal origin. The wastewater samples from these tanks as well as biofilm samples collected from tanks later in the treatment train did not show this pattern ( Figure 9).

The microbial taxonomic composition of the immersed biofilm in the downstream tanks increased in richness with Actinobacteria, Alphaproteobacteria, Bacilli, Gammaproteobacteria, Sphingobacteria and to a lesser degree Betaproteobacteria, and Planctomycetia identified by LCA. This is in contrast to the aqueous phase microbial community, which showed taxonomically rich populations throughout the system with members of the Actinobacteria, Betaproteobacteria, Alphaproteobacteria, and Gammaproteobacteria in greatest abundance ( Figure 9).

The taxonomic composition of the microbial communities forming biofilm on the plant root surfaces immersed in the wastewater changed significantly over the course of the wastewater treatment process. The taxonomic dissimilarity ( Figure 4) observed among the immersed plant root biofilm samples showed three distinct communities: The anoxic (ANOX_B) and the closed aerobic (CLO_B); the first planted aerobic (HR1_B); the second and third planted aerobic tanks (HR2_B and HR3_B); and the sand filter (SAND_B). The changing characteristics of the first tanks of the treatment system (anoxic to aerobic conditions) is likely driving a transition from anaerobes such as *Bacteriodes,* and facultative anaerobes or microaerophiles such as *Bacillus, Clostridium, Nocardia, Mycobacterium,* to aerobic communities. The abundance of the facultative anaerobe group, Bacilli is likely promoted by the oxygen limiting environment in the first two tanks ^43^. The immersed biofilm microbial community of the first planted aerobic tank (HR1) is taxonomically distinct from HR2 and HR3 despite identical physiochemical conditions. This pattern may be influenced by the composition of plant species in the individual tanks, which varies from tank to tank, or perhaps the diminishing influence of organisms contributed by feces.

There is limited ability to relate the results of our analysis of the microbial communities of this ecological WWTP to that of other systems serving different populations and geographic locations as this appears to be the first published findings on the topic. However, metagenomic analyses of the microbial ecology of conventional (activated sludge) wastewater systems have been described elsewhere ^59– 62^.

For example, Lee *et al.,* 2014 ^59^ employed 16S rRNA gene microarrays (PhyloChip) to establish a baseline microbial community structure of the municipal WWTP aeration basin. The microbial taxonomic composition of the aeration basin showed some similarities with that of the entire ecological WWTP sampled here. Specifically, Proteobacteria and Firmicutes were abundant in both the conventional system and ecological WWTP sampled here. To assess the microbial community seasonal variation of activated sludge over a four-year period Ju *et al.*, 2014 ^63^ employed WMS, as was used here. They showed variation in microbial taxonomic composition between the summer and winter samples. They also found variation in the microbial community composition over the four years sampled, irrespective of season. The metabolic structure of activated sludge according to SEED subgroups appeared to remain stable, in spite of variation in taxonomic composition, which suggests microbial community functional redundancy may be present in these systems. The ecological WWTP sampled for this work is housed in a climate-controlled glass house, which raises questions as to whether the microbial community varies from year to year and season to season. The microbial communities are not likely to exhibit temperature-dependent seasonal variation. Taxonomic variation may occur as a result of changes in the microbial communities contributed by the visitors.

The role plants have on influencing the structure of the root-colonizing microbial communities appeared to increase after the first three treatment tanks. The attenuation of taxa contributed by feces ( *Bacillus*) after the first three treatment tanks is reflected in the increased microbial taxonomic heterogeneity in the latter two planted aerobic tanks (HR2 and HR3), which is reflected in the branch lengths for these samples in the neighbor joining network shown in Figure 4. These results indicate the need to design ecological WWTPs with sufficient retention time to allow for the attenuation of stool microbial communities and the development of diverse microbial biofilm communities.

### Removal of PPCPs by the ecological WWTP

The dominant PPCP removal process from the wastewater appears to be partitioning to sludge (biosolids) and biodegradation under nitrifying conditions, which are both reflected in the reductions in aqueous PPCP concentrations that occurred early in the treatment process. Primary sludge settles out of the wastewater in the holding tank and is periodically removed for off-site disposal. The concentration of some of the detected PPCPs continued to decline as the wastewater continued through the system ( Table 1) indicating some continued removal beyond the first two or three tanks. For example, while the concentration of caffeine declined between the holding tank (9.5 × 10 ^4^ ng L ^-1^) and the anoxic tank (1.9 × 10 ^4^ ng L ^-1^), further reduction from the aqueous phase was observed in the subsequent three aerobic treatment tanks (CLO, HR1, HR2). Given that caffeine is a hydrophilic organic base (low K _OW_) only moderate partitioning to sludge is expected (86) and microbial biodegradation is likely to be responsible for the reduction in caffeine concentrations observed from the aerobic treatment tanks. Ibuprofen concentrations followed a pattern similar to caffeine’s in that significant reductions were seen in the first three aerobic treatment tanks (1.1 × 10 ^4^ ng L ^-1^ to 5.6 × 10 ^2^ ng L ^-1^). Carbamazepine, DEET, and trimethoprim concentrations remained stable throughout the treatment process. The combination of low partition to primary sludge expected and metabolic recalcitrance accounts for their stability in system ^47, 50, 55^.

Microbial biodegradation pathways for most PPCPs have not been characterized, which makes it difficult to directly detect responsible genes. Nevertheless, ammonia oxidizing bacteria have been associated with the biodegradation of some PPCPs, while others have been shown to be degraded by nitrite oxidizing bacteria ^64, 65^. The relative abundances of sequences that MEGAN associated with ammonia monooxygenases were very low throughout the system (ranging from 0 to 53 reads), but were highest in the biofilm samples obtained from the HR3 and HR2 tanks. Due to lower dissolved oxygen levels, genes involved in denitrification (nitrite reductases) were found to be in highest relative abundance in the anoxic (ANOX) and closed (CLO) tanks (644 and 688, respectively).

Functional attributes of detected taxa reported in the literature can be used to identify metabolic potential pertinent to uncharacterized xenobiotic metabolic pathways. For example, in the first three tanks, the Firmicutes colonizing plant root surfaces have been reported to metabolize xenobiotics. *Bacillus cereus, B. megaterium* and *B. amyloliquefaciens* have been reported to metabolize phenol ^66^, crude oil ^67^, textile dyes ^68^, and other xenobiotics through the induction of cytochrome P450s ^69, 70^. *Dehalobacter* sp. FTH1, identified in the plant root biofilm sample obtained from the second planted aerobic tank (HR2), has been reported to dechlorinate a number of organohalide xenobiotics ^71, 72^. *Clostridium*, identified in the root biofilm sample obtained from the first aerobic tank (HR1), has been reported to be involved in metabolism of bromophenols as a member of a consortium including *Delhalobater* ^73^. *Entercoccus* spp., identified in the plant root biofilm of HR2, has been reported to degrade azo dyes ^74^.

Of the Actinomycetales identified, *Mycobacterium* spp., which have been reported to metabolize a variety of xenobiotics including polycyclic aromatic hydrocarbons ^75^, biphenyls ^76^, as well as various pharmaceuticals ^77^ were identified in low relative abundance in samples obtained from the biofilm growing on plant roots in the anoxic tank and in high relative abundance in the biofilm sampled from sand filter. *Mycobacterium* spp. was also identified in the aqueous wastewater throughout the system ( Figure 2 & Figure 9). While this metabolically plastic genus has been reported to be capable of metabolizing a wide variety of xenobiotics it should be noted that there are numerous pathogenic taxa including *M. tuberculosis*, *M. bovis*, and *M. avium* among others. The biofilm sample obtained from the sand filter contained 10,282 reads associated with human diseases and 18,766 reads associated with xenobiotic metabolism by the LCA algorithm in MEGAN.

Rhizobiales, which were identified in the aqueous phase throughout the system as well as in the biofilm sampled in the latter three treatment tanks (HR2, HR3, and SAND) have been reported as abundant in biofilm reactors treating sulfamethoxazole containing wastewater ^78^. Also present throughout the system’s aqueous phase were members of the Rhodobacteraceae, Burkholderiaceae, Comamonadaceae, *Pseudomonas*, and Xanthomonadaceae, which have been reported to metabolize aromatic hydrocarbons ^79, 80^. Among these, the genus *Pseudomonas* has been identified as capable of biodegradation a variety of xenobiotic including some pharmaceuticals in a number of other settings including membrane bioreactors ^81^, cultures originating from pharmaceutical wastewaters ^82^, and environmental samples ^83^.

Greater xenobiotic metabolic heterogeneity was observed in the samples obtained from the plant root-associated biofilm as compared to the free-floating aqueous microbial community. The aqueous microbial metagenome, collectively, contained a greater total for xenobiotic metabolism gene copies (1.2 × 10 ^5^ compared to 8.6 × 10 ^4^ for the plant root biofilm) (see Supplementary material ST5). When comparing the proportion of sequences identified in the aqueous and biofilm phases of the system, which represent the non-root-associated and root-associated microbial populations, respectively, the xenobiotic metabolism gene categories nitrotoluene, benzoate, flurobenzoate and steroid degradation were found to be significantly higher in the aqueous phase samples (Welch’s t-test p-values < 0.05) (see Supplementary material ST49) ( Figure 7). However, when comparing the type and abundance of reads associated with xenobiotic metabolism (KEGG level 3) the aqueous phase samples all resembled one another whereas, the biofilm samples were heterogeneous (see Figure 7 and Supplementary material ST31).

For aqueous phase microbial communities, growth of microbial consortia with the capacity to metabolize a given PPCP is driven by the presence of the compound in the wastewater, which will fluctuate with time. Stationary biofilm communities are likely to be more stable populations. These communities can accumulate with time and potentially acquire metabolic genes by horizontal gene transfer ^84^. In contrast to the aqueous phase xenobiotic metabolism, which was dominated by benzoate degradation genes, the plant root microbial biofilm metagenomes contained higher relative abundances of other categories including aminobenzoate, chloroalkane/chloroalkene, and to a lesser degree ethylbenzene categories ( Figure 7). However, of these categories, only chloroalakane/chloroalkene was significantly higher (Welches t-test p-value 0.031) in the biofilm samples collectively (see Supplementary material ST49).

While others have identified ammonia oxygenases and nitrite reductases as being involved in microbial PPCP biodegradation ^64^, the relative abundances of these gene categories were very low throughout the system. The relative abundance of xenobiotic metabolism gene copies was highest for the sand filter samples at 18,318 for SAND_W and 18,766 for SAND_B (see Supplementary material ST30). The sand filter’s ability to attenuate and accumulate sloughed off microbial cells as the wastewater passes through may be driving an accumulation of microbial biomass. If this is the case, sand filters are likely to have populations of the microbial communities found throughout the aqueous phase of the system, yet may not serve as a location of high metabolic activity, thereby contributing little to the metabolism of xenobiotics. The increase in concentrations observed for some of the PPCPs (caffeine, carbamazepine, DEET, gemfribozil, and ibuprofen) after the wastewater passed through the sand filter, if a real trend, could support this perspective.

Culture bias was reflected in the taxonomic composition of the enrichment cultures growing on the sole pharmaceutical carbon sources examined here. While culture bias is well known ^85^ and was expected, the enrichment of organisms capable of metabolizing individual pharmaceutical compounds from the WWTP effluent water reflects the ability for the ecological WWTP to support this metabolic capability. Only one taxon ( *Bacillus cereus*) was identified in at least one enrichment culture (carbamazepine) and the biofilm sampled from the WWTP and one taxon ( *Mycobacterium* spp.) was identified in the aqueous, biofilm and carbamazepine enrichment cultures ( Figure 6). Given the selective pressure supplied by culturing, it is unlikely that these two taxa are solely responsible for the biodegradation of carbamazepine in the ecological WWTP. However, having isolated pharmaceutical metabolizing consortia from effluent water suggests the ecological WWTP supports microbial populations with the capacity remove recalcitrant micropollutants from wastewater.

Secondary plant metabolites contributed to the wastewater by the tropical species cultivated in planted tanks may help support populations of microbial communities with xenobiotic degradation capabilities by providing a more consistent supply of structurally diverse carbon sources. The most abundant category of xenobiotic metabolism genes was associated with benzoate degradation in nearly all the samples. Benzoate degradation is known to play a role in the degradation of a variety of aromatic compounds ^23, 86, 87^. The dominance of this functional category suggests that microbial communities in the aqueous phase were metabolizing a variety of benzoate-containing compounds, which were likely to include metabolites of plant and xenobiotic origin. However, further work is needed to determine whether plant-microbe feedback processes promote PPCP biodegradation in ecological WWTPs.

## Data availability

The data referenced by this article are under copyright with the following copyright statement: Copyright: © 2016 Balcom IN et al.

FASTQ files and associated metadata are available at NCBI BioProject ID PRJNA286671 ( http://www.ncbi.nlm.nih.gov/bioproject/286671).

## Supplementary material

**Data tables ST1–49**
