# Supplementary material for: Metagenomic analysis of an ecological wastewater treatment plant’s microbial communities and their potential to metabolize pharmaceuticals
Source: F1000Res. 2016 Jul 28;5:1881. [Version 1] doi: 10.12688/f1000research.9157.1 (PMC4995686; doi:10.12688/f1000research.9157.1)

Supplementary material for Balcom et al. 2016

Figures S1-S22

Caption for Figures S1-S2:

Phylograms depicting the lowest common ancestor taxonomic profile for Human Microbiome Project (HMP) control sample 902 and CosmidID’s constructed freshwater sample from the Delaware River generated in MEGAN 5.7.10 software. The number of reads associated with each taxonomic classification is shown. The minimum-support percent threshold in MEGAN analyses was set to 1.0 %.

Caption for Figures S3-S14:

Phylograms depicting the lowest common ancestor taxonomic profile for each of the WWTP samples generated in MEGAN 5.7.10 software. The notation used to indicate aqueous and immersed biofilm samples is _W and _B, respectively. The number of reads (normalized) associated with each taxonomic classification is shown. The minimum-support percent threshold in MEGAN analyses was set to 1.0 %.

Caption for Figures S15-S22:

Phylograms depicting the lowest common ancestor taxonomic profile for each of the sole pharmaceutical compound carbon source enrichment culture samples generated in MEGAN 5.7.10 software. The notation used to indicate cultures grown on carbamazepine, trimethoprim, and sulfamethoxazole carbon sources is C, T, and S, respectively, and trailing numbers indicate replicate number. The number of reads (normalized) associated with each taxonomic classification is listed to right of each terminal taxon. The minimum-support percent threshold in MEGAN analyses was set to 1.0 %.

Figure S1

HMP 902
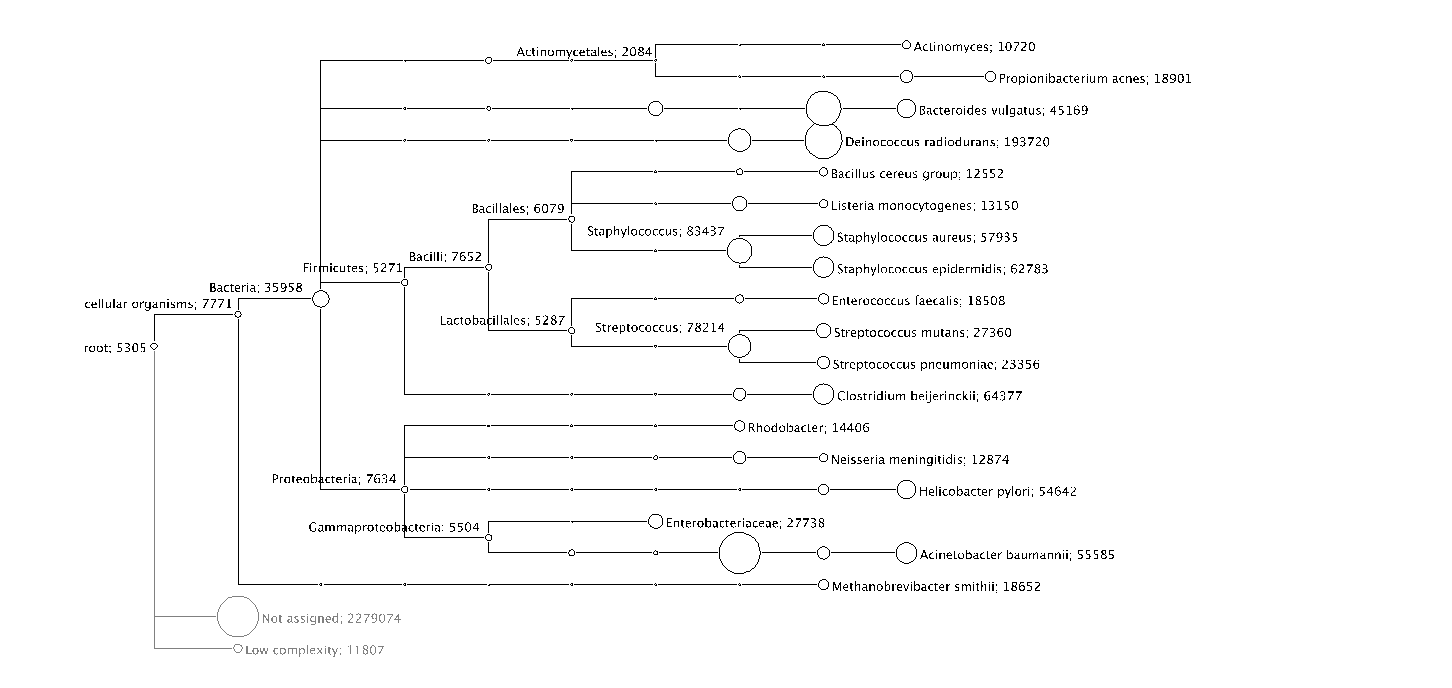


Figure S2

Delaware River


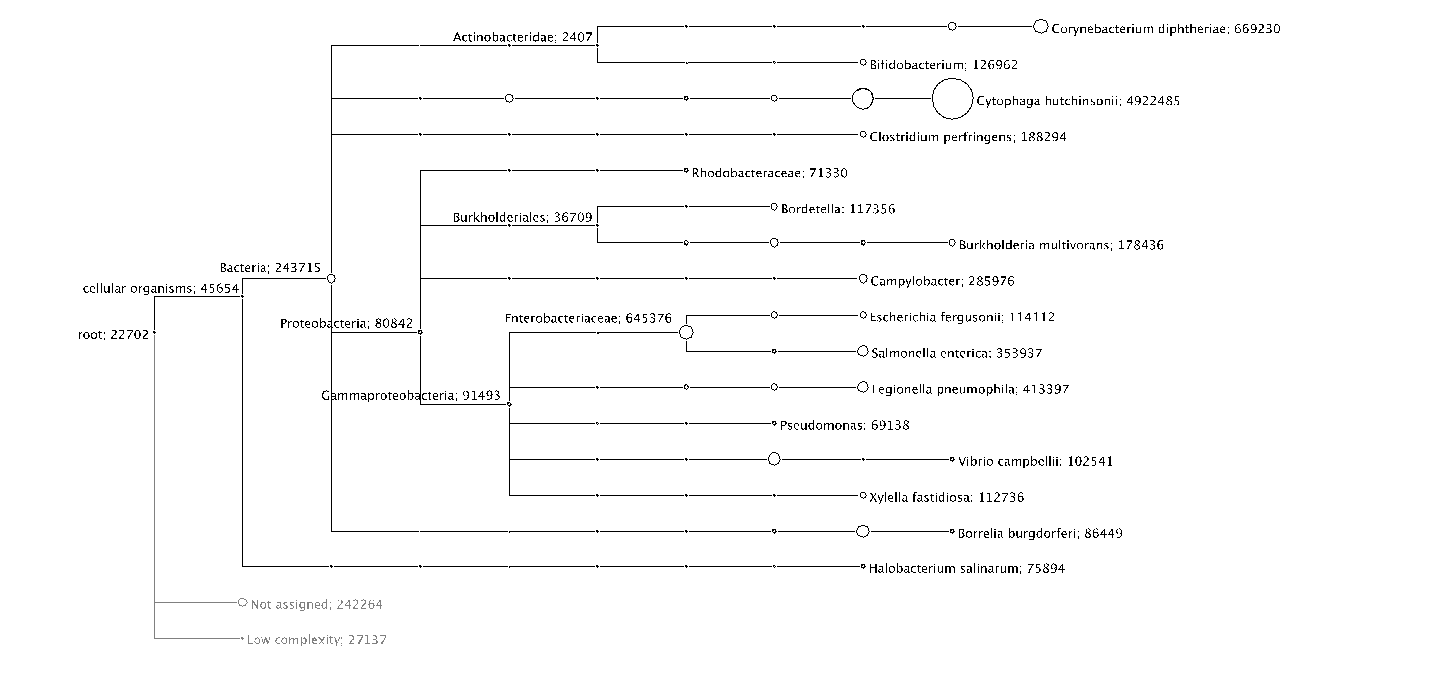


Figure S3

ANOX_B


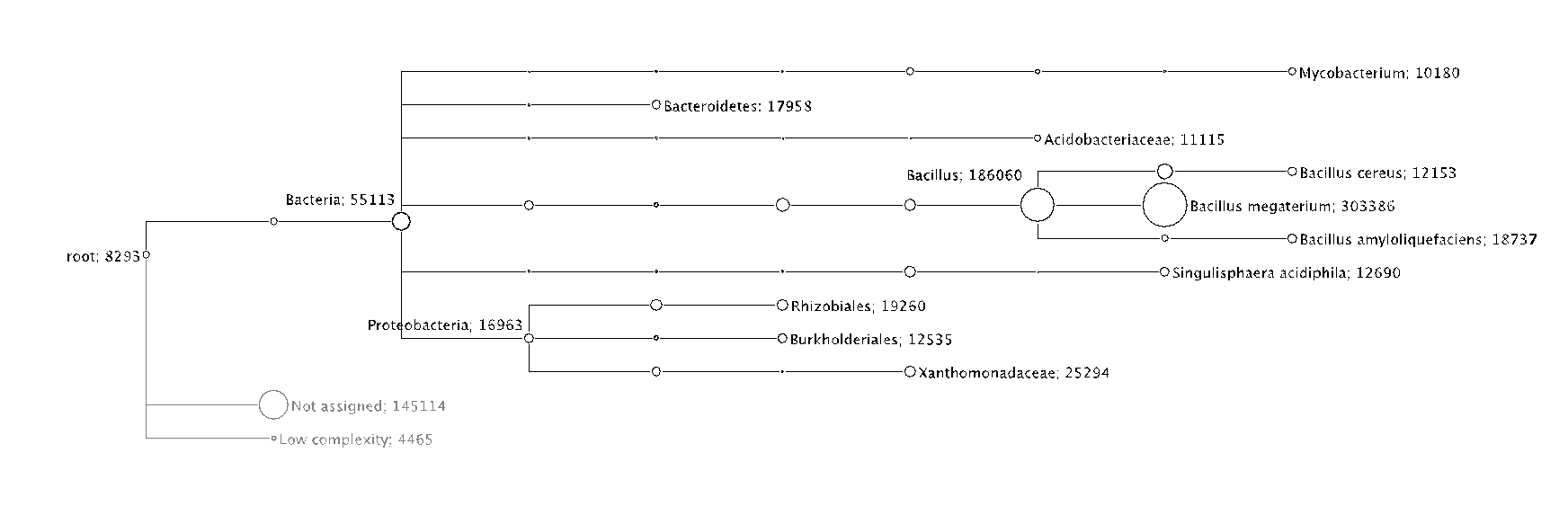


Figure S4

ANOX_W


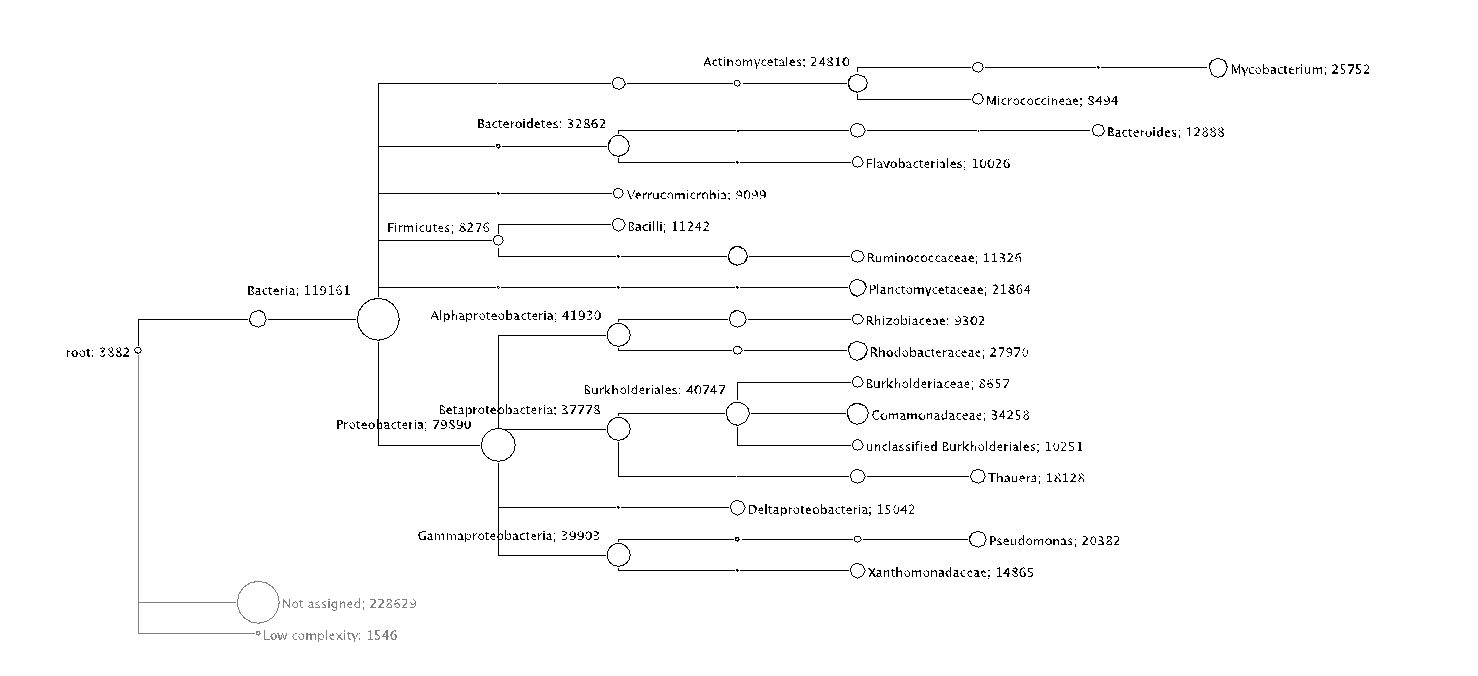


Figure S5

CLO_B


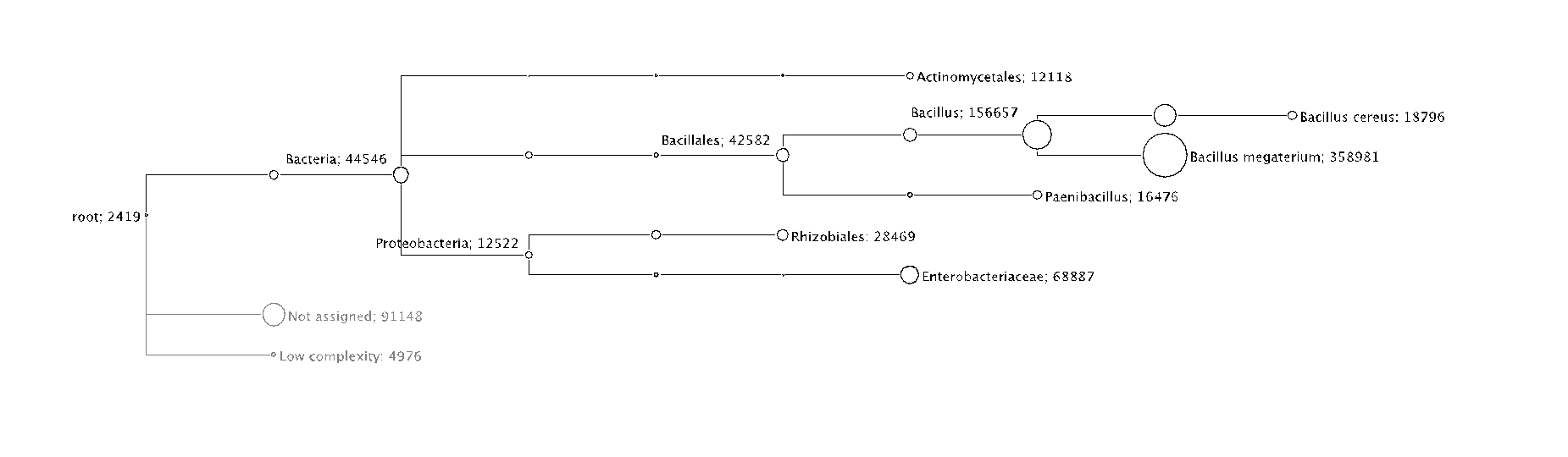


Figure S6

CLO_W


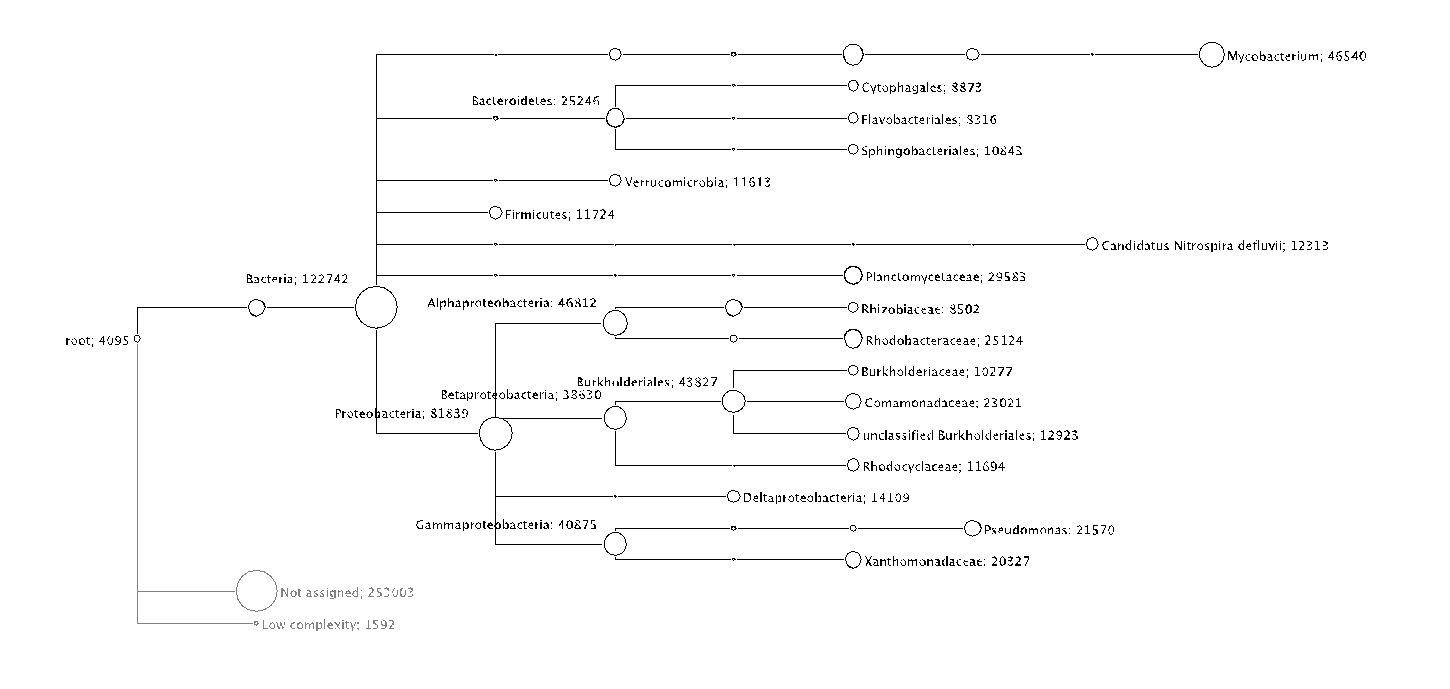


Figure S7

HR1_B


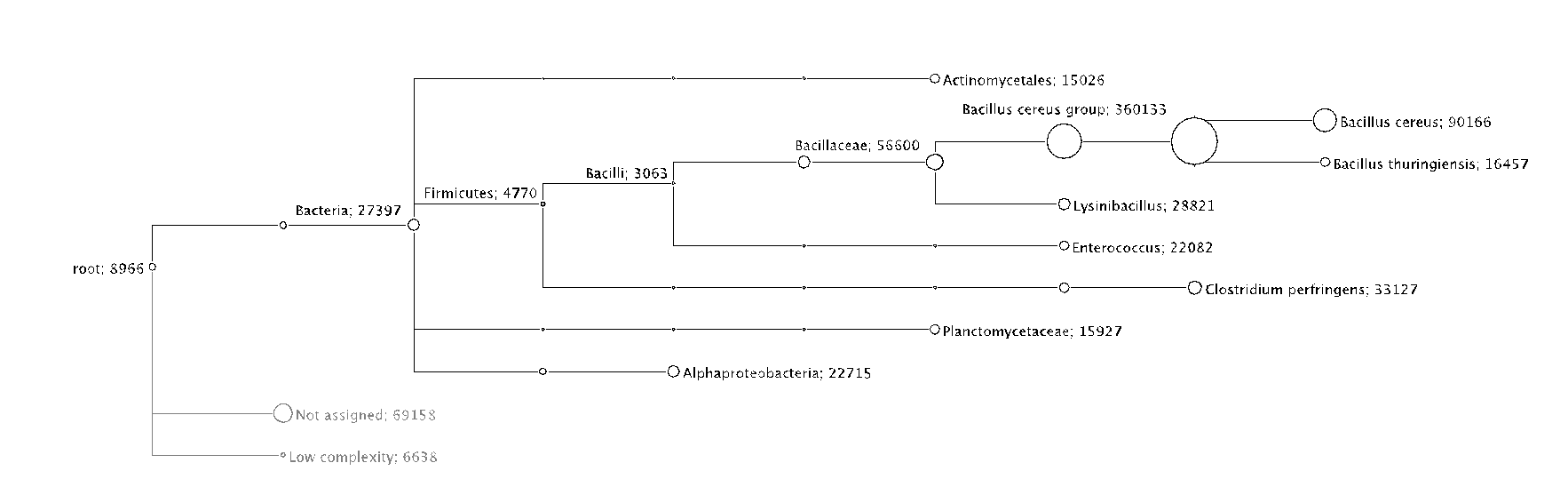


Figure S8

HR1_W


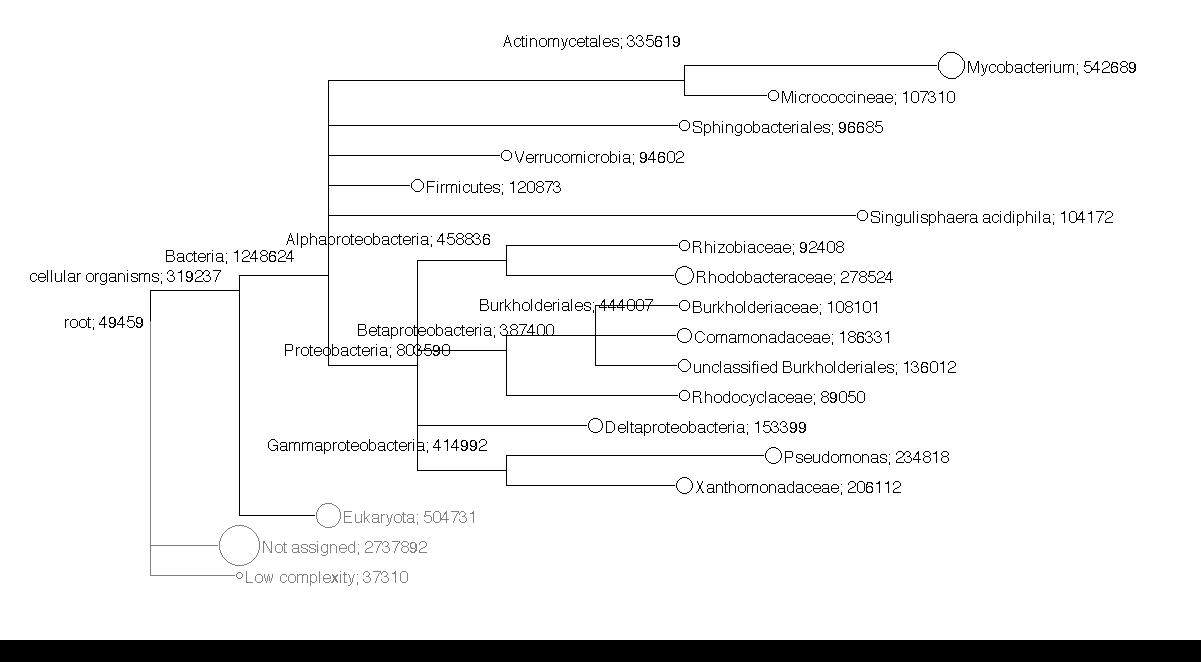


Figure S9

HR2_B


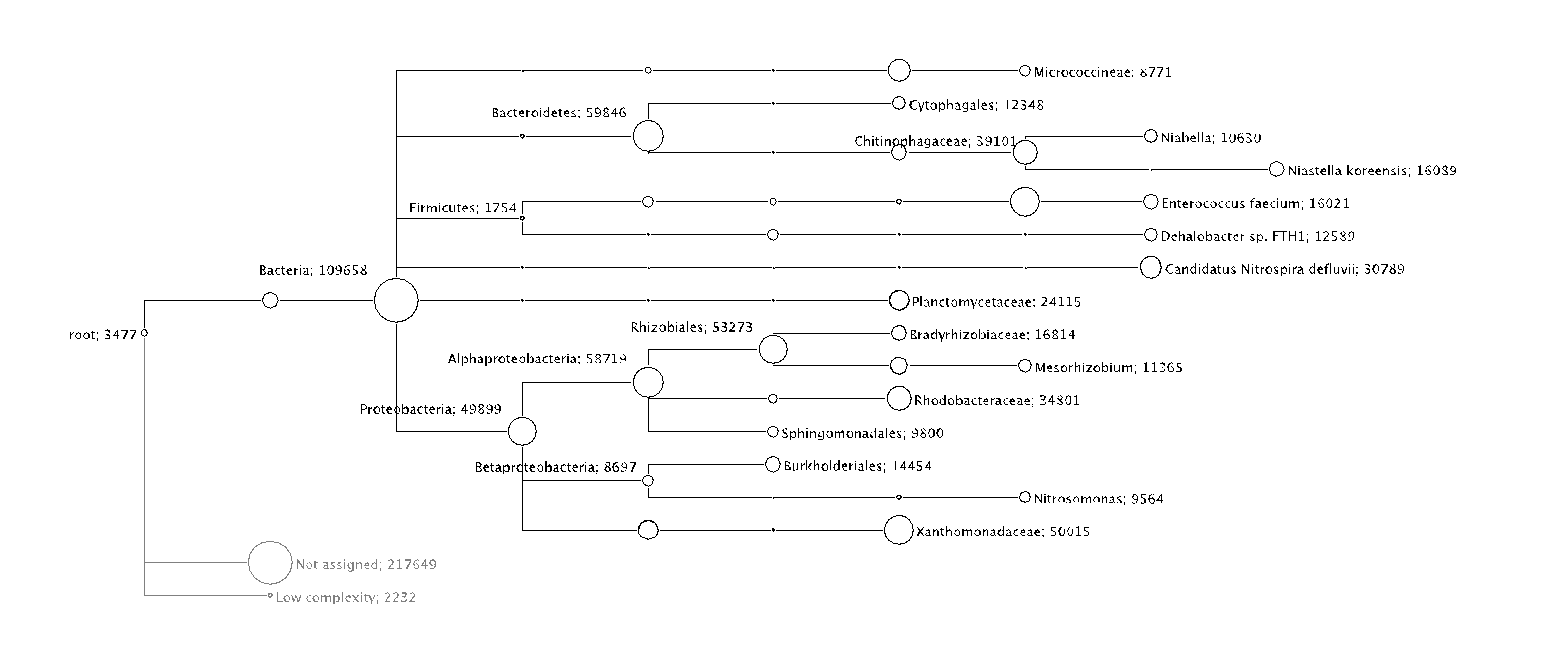


Figure S10

HR2_W


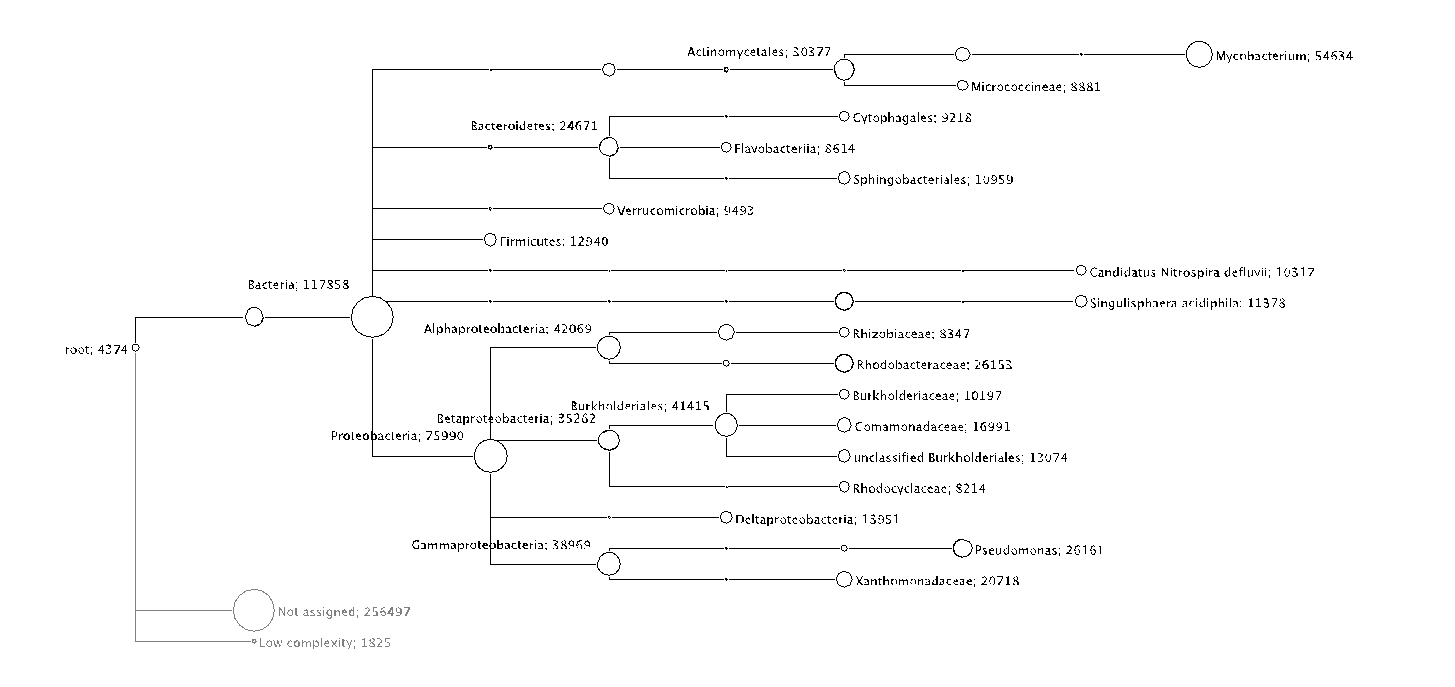


Figure S11

HR3_B


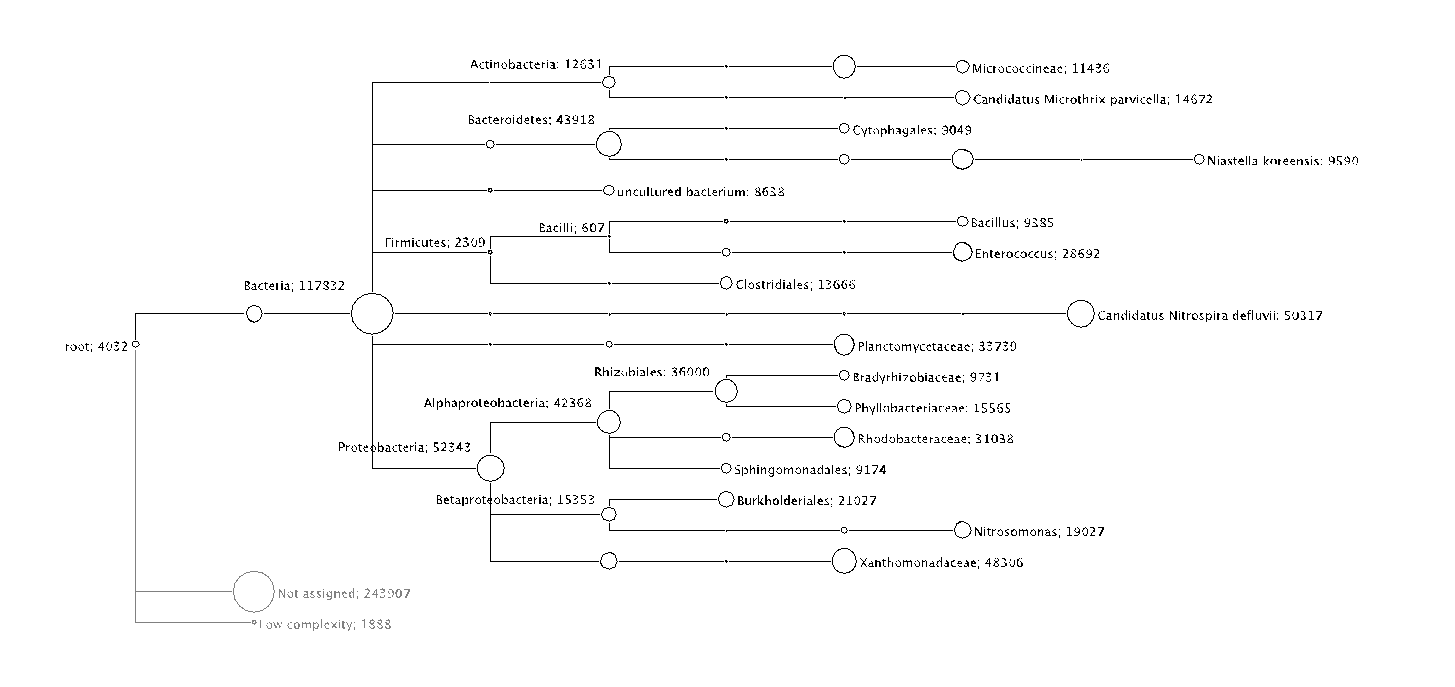


Figure S12

HR3_W


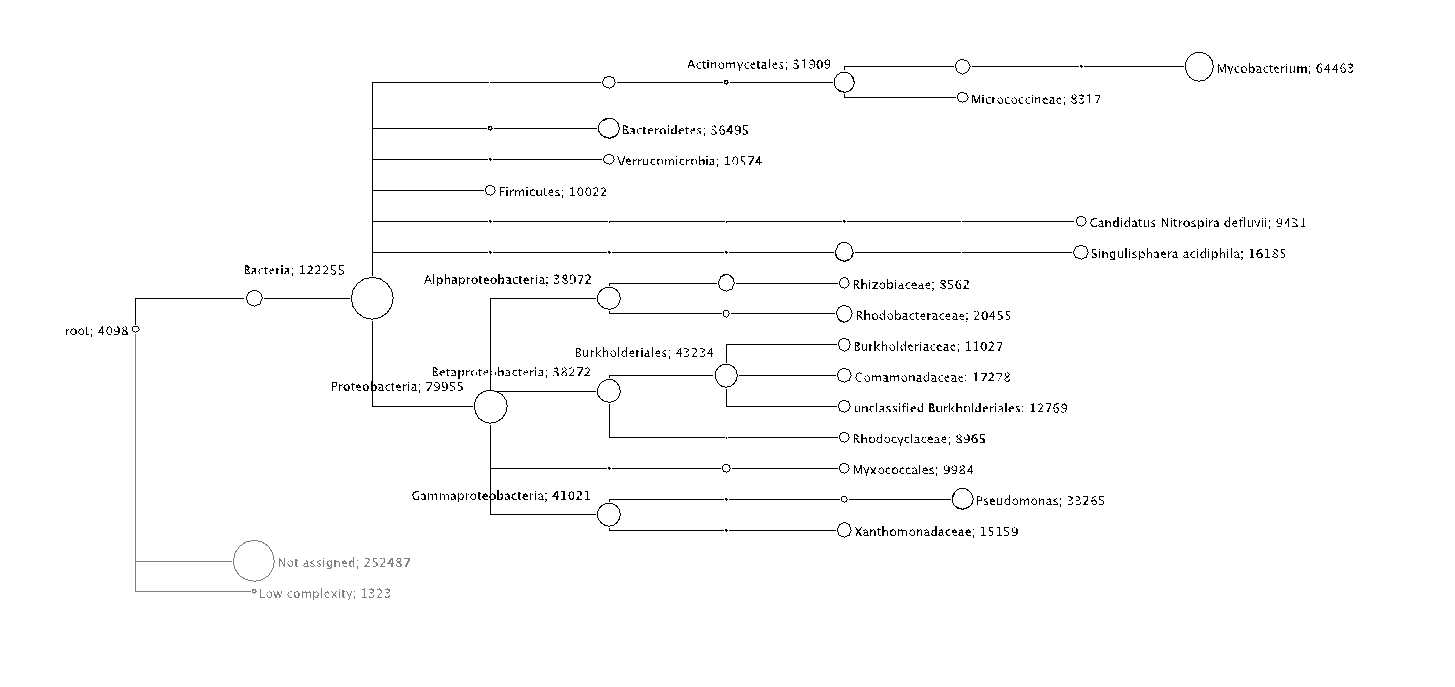


Figure S13

SAND_B


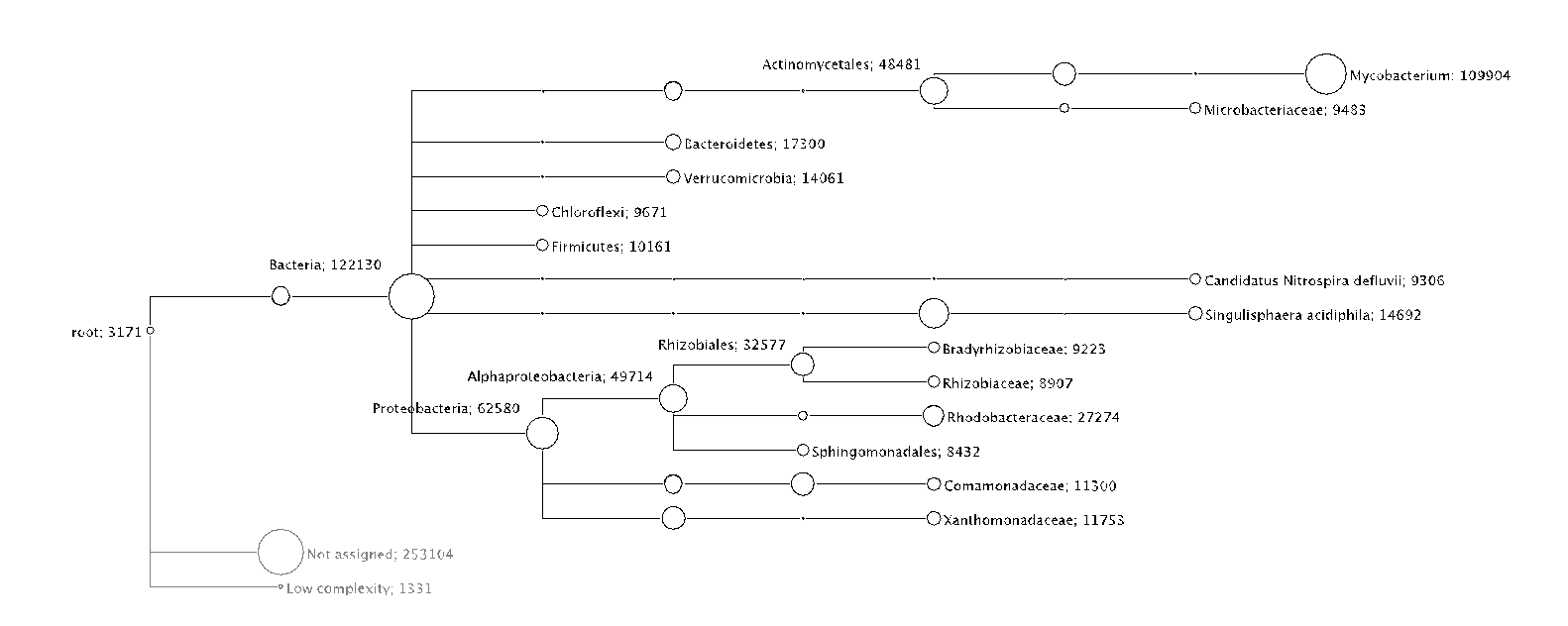


Figure S14

SAND_W


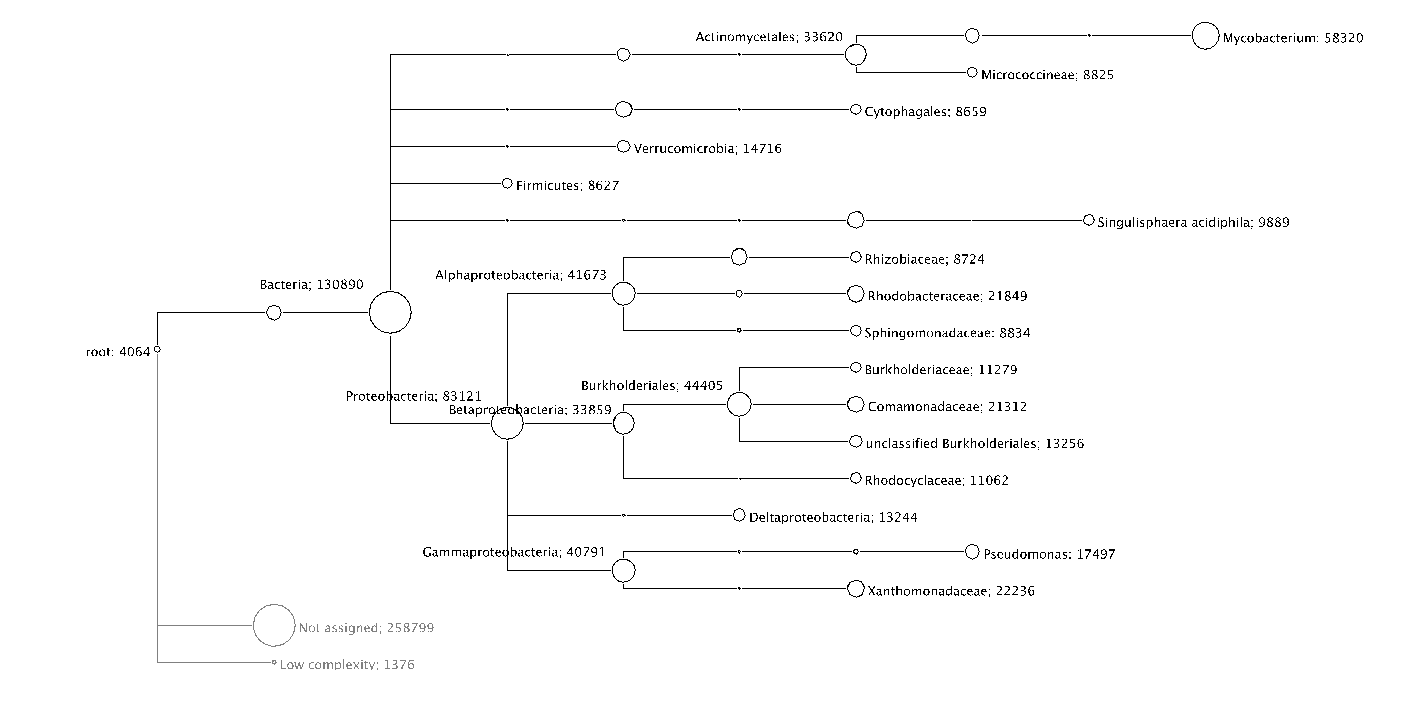


Figure S15

C3A


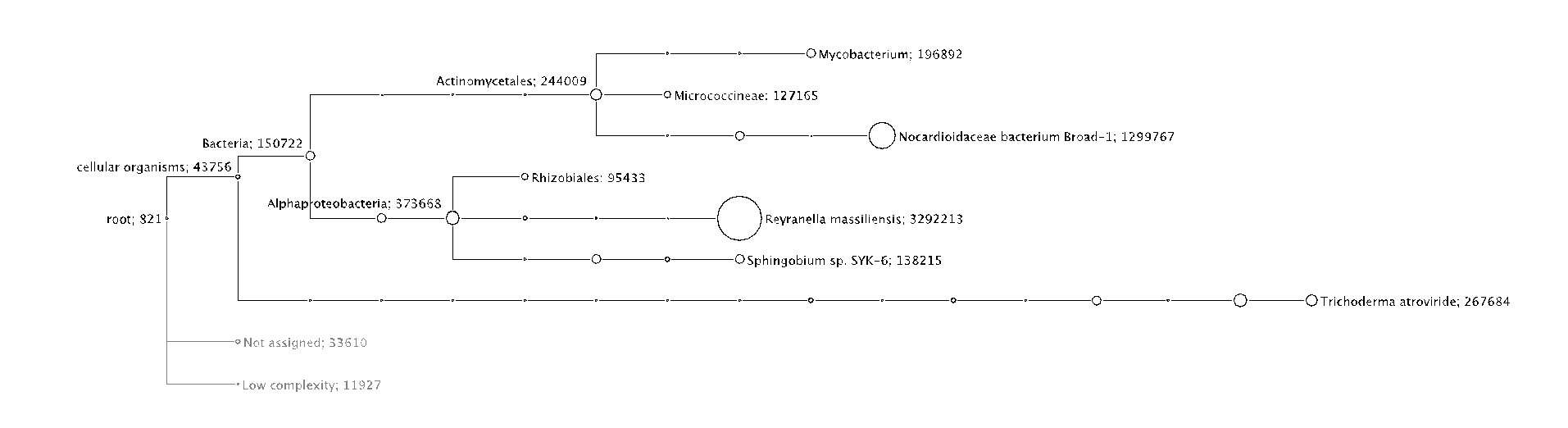


Figure S16

C3B


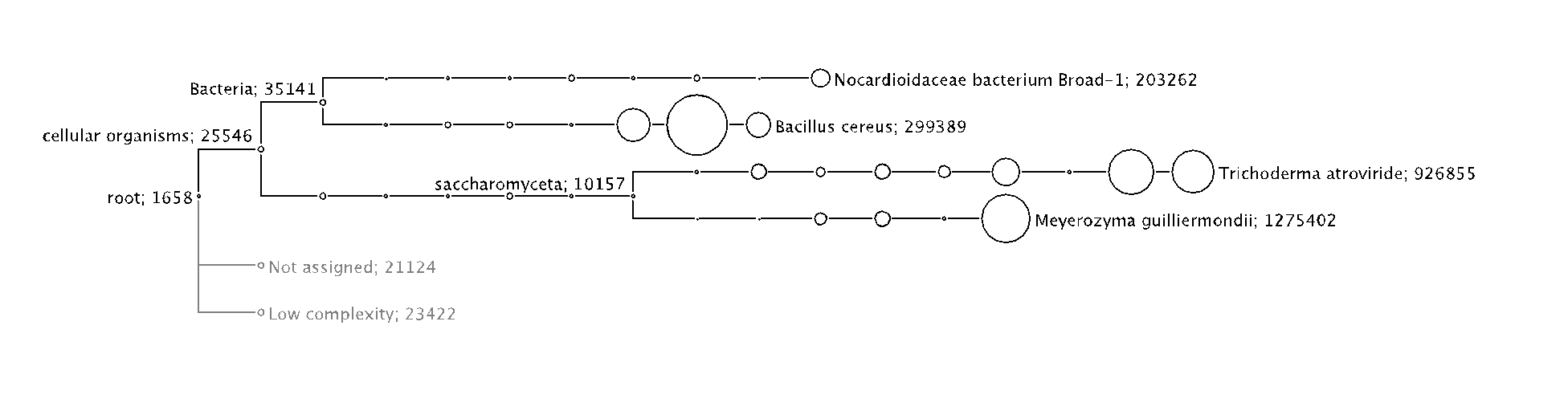


Figure S17

C3D


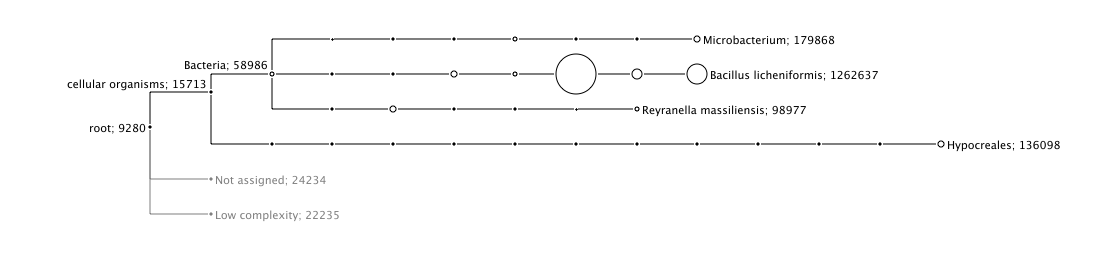


Figure S18

S3B


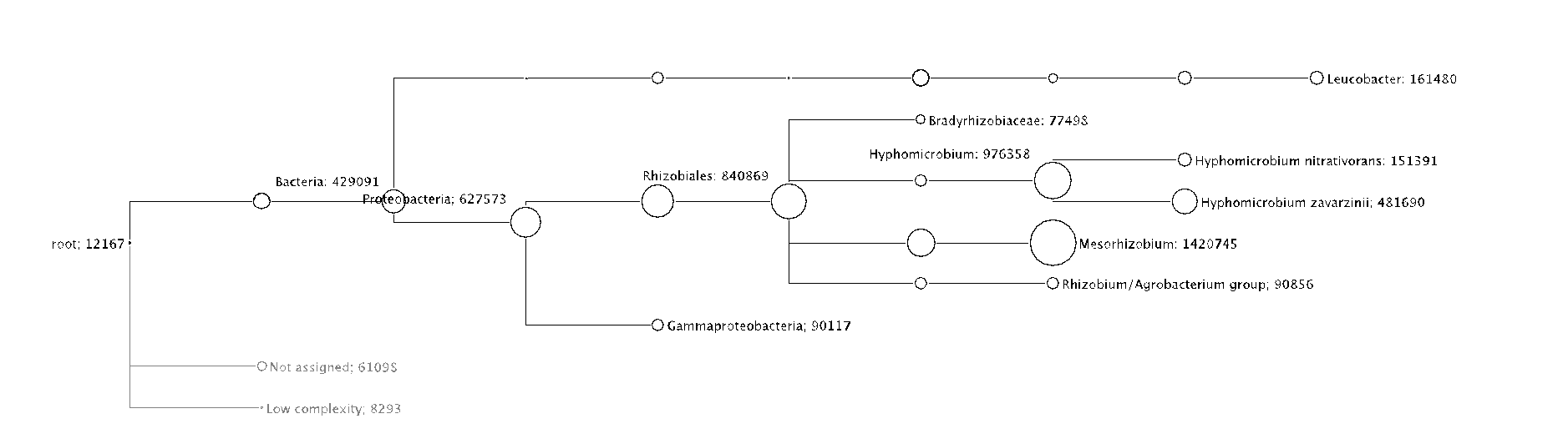


Figure S19

S3D


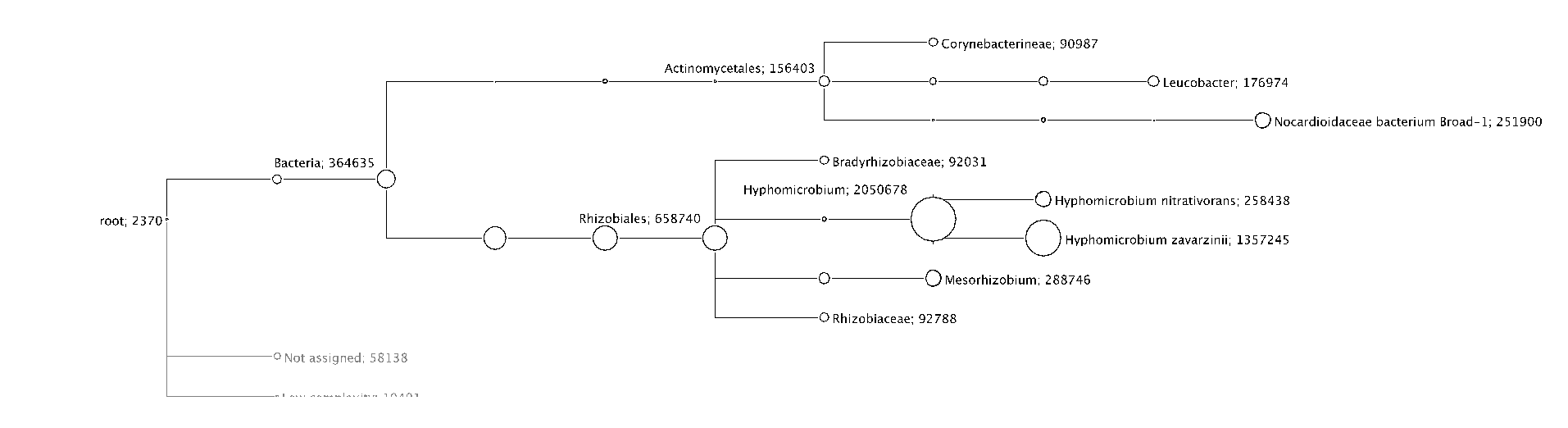


Figure S20

T3B


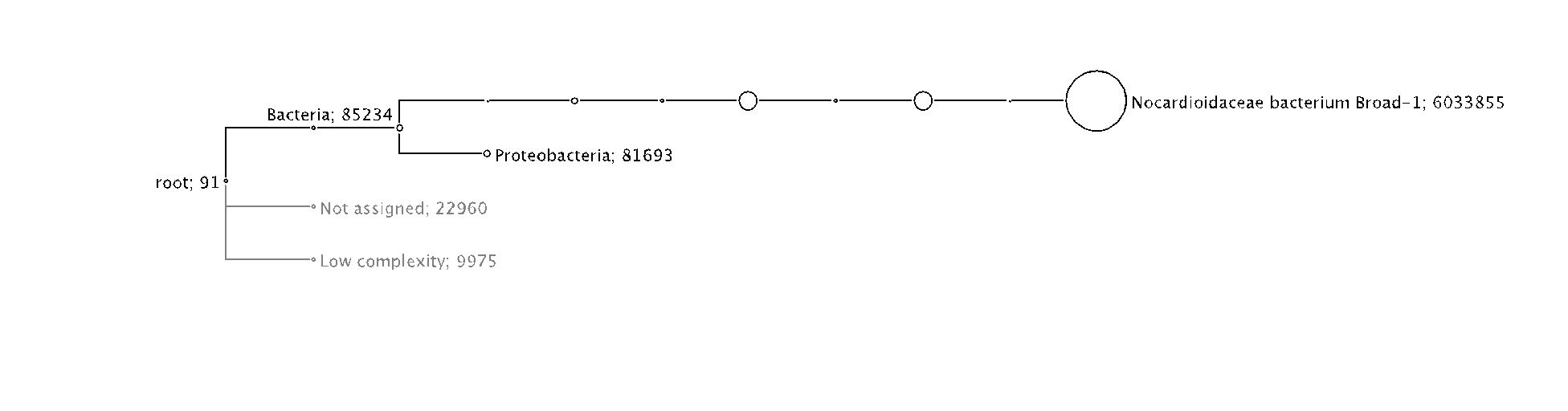


Figure S21

T3C
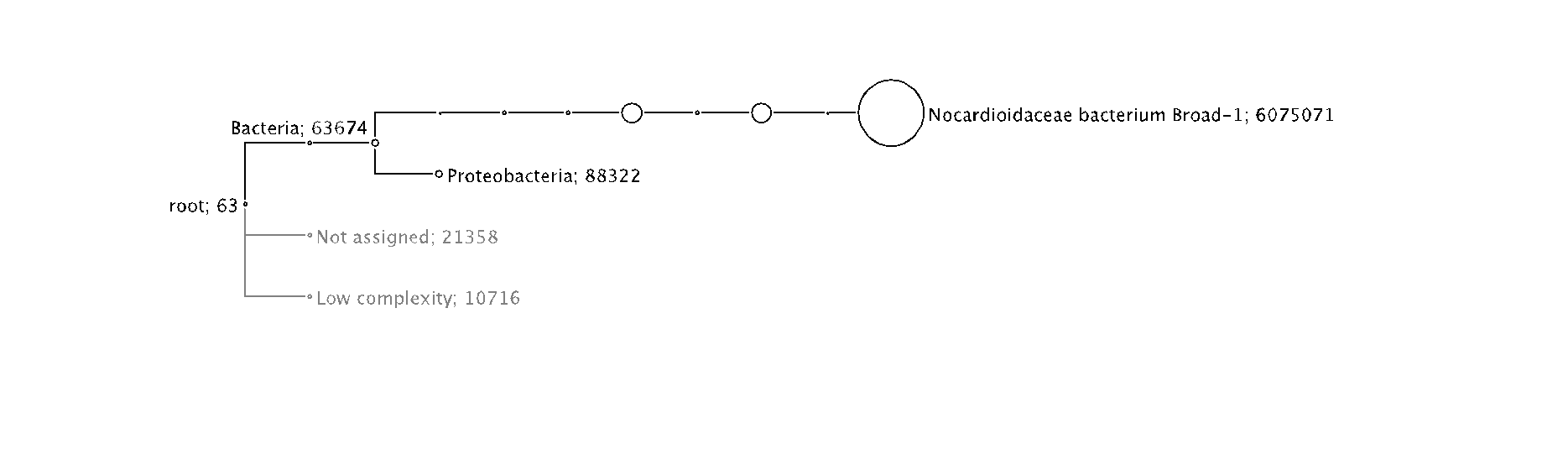


Figure S22

T3D


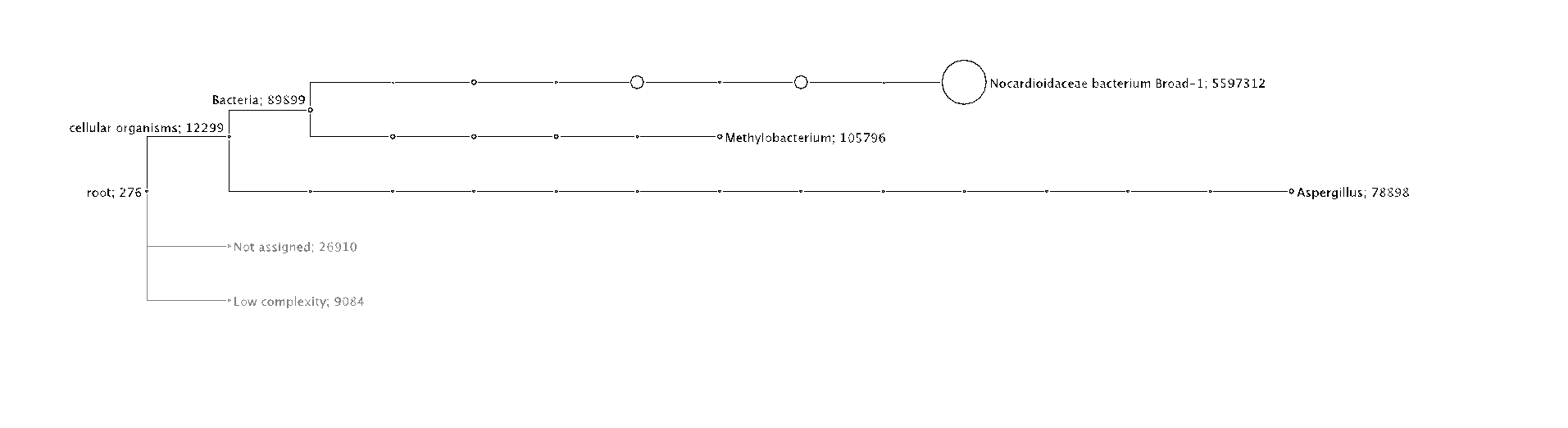

Supplement: Supplementary file 2 [file f1000research-5-9854-s0001.tgz › 753e7434-006e-4171-9fac-0519c4de4af8.docx]
